# Mitochondrial genome evolution in the *Saccharomyces sensu stricto* complex

**DOI:** 10.1371/journal.pone.0183035

**Published:** 2017-08-16

**Authors:** Jiangxing Ruan, Jian Cheng, Tongcun Zhang, Huifeng Jiang

**Affiliations:** 1 College of Biotechnology, Tianjin University of Science & Technology, Tianjin 300308, China; 2 Key Laboratory of Systems Microbial Biotechnology, Tianjin Institute of Industrial Biotechnology, Chinese Academy of Sciences, Tianjin, China; Institut de Genetique et Microbiologie, FRANCE

## Abstract

Exploring the evolutionary patterns of mitochondrial genomes is important for our understanding of the *Saccharomyces sensu stricto* (*SSS*) group, which is a model system for genomic evolution and ecological analysis. In this study, we first obtained the complete mitochondrial sequences of two important species, *Saccharomyces mikatae* and *Saccharomyces kudriavzevii*. We then compared the mitochondrial genomes in the *SSS* group with those of close relatives, and found that the non-coding regions evolved rapidly, including dramatic expansion of intergenic regions, fast evolution of introns and almost 20-fold higher rearrangement rates than those of the nuclear genomes. However, the coding regions, and especially the protein-coding genes, are more conserved than those in the nuclear genomes of the *SSS* group. The different evolutionary patterns of coding and non-coding regions in the mitochondrial and nuclear genomes may be related to the origin of the aerobic fermentation lifestyle in this group. Our analysis thus provides novel insights into the evolution of mitochondrial genomes.

## Introduction

Mitochondria (MT) are one of the two endosymbiotic organelles with non-nuclear genetic materials found in eukaryotic cells [1]. Although most of the genes from ancestral mitochondria have been transferred into the nuclear genome [2], mitochondrion are essential for cell respiration [3], biosynthesis of certain metabolites [4], ion homeostasis and apoptosis [5]. The *Saccharomyces sensu stricto* (*SSS*) group, which includes *S*. *cerevisiae*, has been used for a long time to study the evolutionary patterns of mitochondrial genomes [6]. Significantly, members of the *SSS* group can survive on fermentable carbon sources even in the absence of mitochondrial DNA (mtDNA) [7], due to the appearance of the aerobic fermentation lifestyle after a whole-genome duplication event (WGD) [8]. The mitochondrial genome size in the *SSS* group has expanded two- to four-fold compared to close relatives [9–11], even though they diverged only ~10–20 million years ago (MYA) [12]. The wide diversity of mitochondrial genomes in the *SSS* group thus provides an excellent case for investigating the mechanisms of genome evolution among (closely-related) species [13–16].

Due to the development of next-generation sequencing technologies, several genomes of species from the *SSS* group are now available [17]. The mitochondrial genome of *S*. *cerevisiae* was first sequenced in 1998 [18]. However, the mtDNA sequence of most species in the *SSS* group is still unclear, with the exception of *S*. *cerevisiae*, *S*. *paradoxus* [19] and *S*. *uvarum* [20]. In this study, we obtained the complete mtDNA sequences of *S*. *mikatae* and *S*. *kudriavzevii* by high-throughput sequencing and bioinformatics analysis to further clarify the evolutionary mechanisms driving mitochondrial genome change in the *SSS* group. We then analyzed the evolutionary processes governing the intergenic regions, coding genes, introns and genomic structures of these newly sequenced mitochondrial genomes. Our results provide new details on the evolution of mitochondrial genomes in yeasts, and general insights into the mechanisms of genomic evolution in eukaryotes.

## Results

### Assembly of the MT genomes of *S*. *mikatae* and *S*. *kudriavzevii*

**Fig 1:**
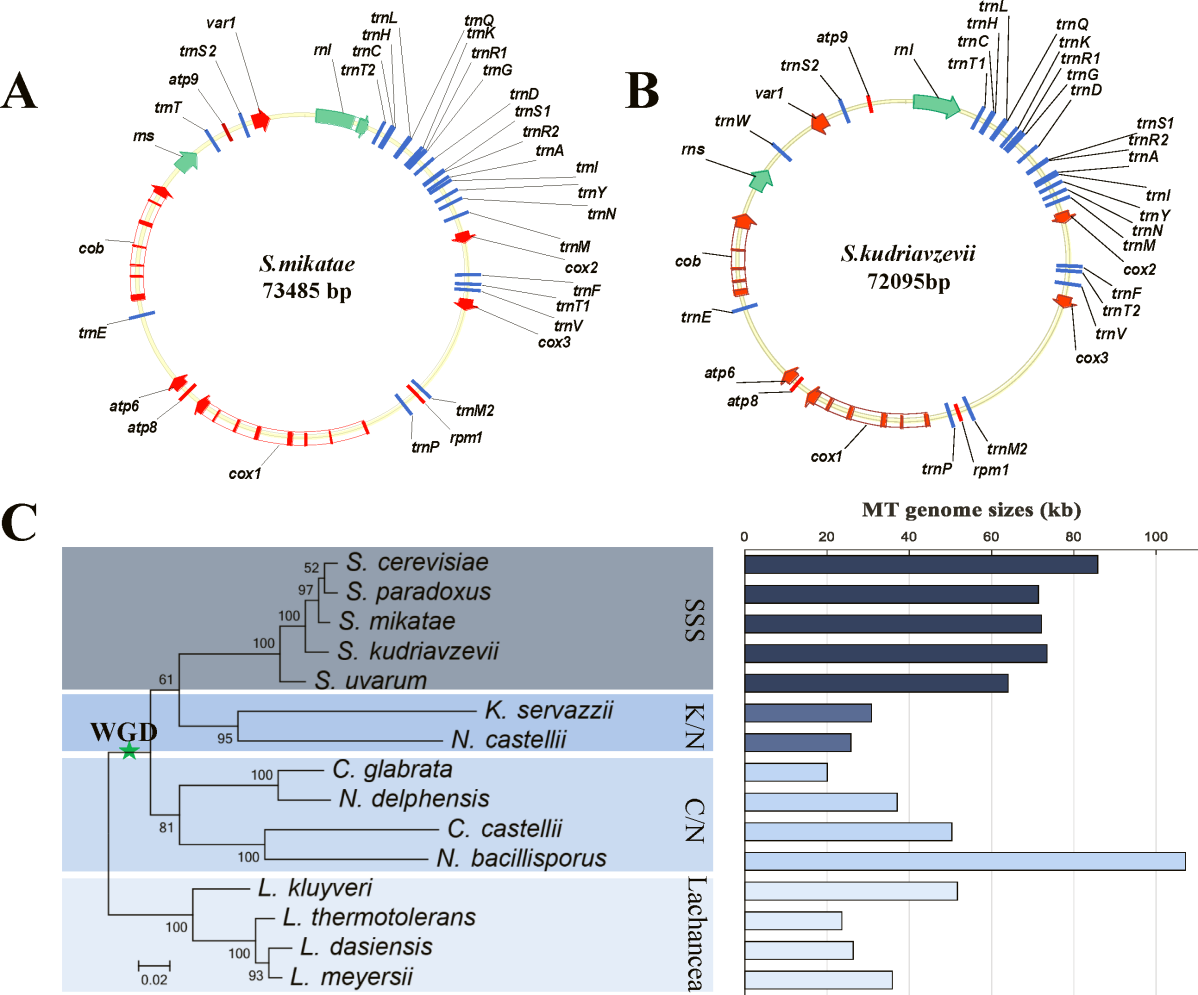
(A) Sequence and assembly-derived map of the mitochondrial genome of *S*. *mikatae*. The proteins and rRNAs are indicated by red and green arrows, respectively, introns are marked with white rectangles and tRNAs with blue bars. (B) Map of the mitochondrial genome of *S*. *kudriavzevii*. (C) Evolutionary tree of the *Saccharomyces sensu stricto* groups and their relatives. The tree was constructed based on the concatenation of eight protein-coding genes in all mtDNAs. The right pane of the histogram shows the size of each MT genome.

To investigate the evolution of MT genomes in the *SSS* group, we first needed to decipher the MT genomes of the species in this clade. The MT genome of *S*. *mikatae* IFO1815 was therefore sequenced and assembled (Fig 1A, Materials and Methods). We then obtained the complete mtDNA sequences of *S*. *kudriavzevii* IFO1802 by assembling fragments from published data [21] and using polymerase chain reaction (PCR) to fill gaps between the fragments (Fig 1B) (see Materials and methods). Similar to the other sequenced species in the *SSS* group, both mtDNA genomes contained a highly conserved functional gene set, which consists of 35 genes encoding the components of cytochrome oxidase (*cox1*, *cox2* and *cox3*), cytochrome b (*cob*), 3 subunits of ATPase (*atp6*, *atp8* and *atp9*), two ribosomal RNA subunits (*rnl* and *rns*), one ribosomal protein gene (*var1*), the *rpm1* gene for the RNA subunit of RNase P, and 24 tRNA genes (S1 Table). Based on a phylogenetic tree of five *SSS* yeasts and ten yeasts from outside of the *SSS* group, we found that the MT genome sizes of the *SSS* group were visibly larger than those of most close relatives (Fig 1C and S2 Table), such as species in the *Kazachstania/Naumovozyma* (K/N), *Candida/Nakaseomyces* (C/N) and *Lachancea* lineages, except for *N*. *bacillisporus* in the C/N linage where the genome was enlarged by the invasion of palindromic GC clusters [22]. Mitochondrial genome expansion in the *SSS* group is mainly caused by the expansion of intergenic regions [6]. A comparison with *C*. *glabrata*, which has minimum intergenic regions, showed that the intergenic regions accounted for 95% of the genome size variation (S1A Fig), including intergenic ORFs, origin of replication (*ori*) sequences, AT spacers and GC clusters (S1A File, S1–S3 Figs and S3–S5 Tables)[23].

### Expansion and evolution of introns

**Fig 2:**
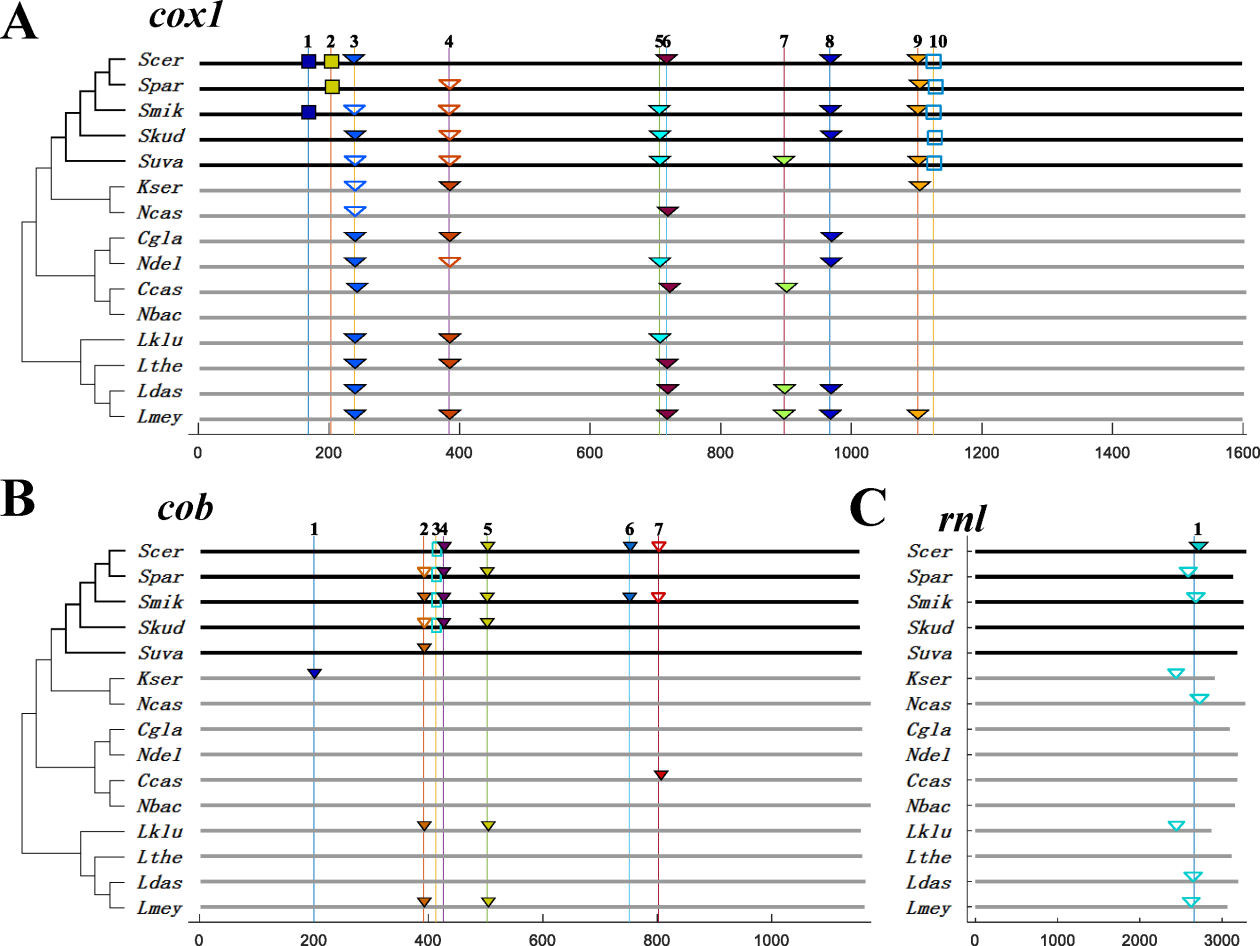
The distribution of introns in mtDNAs. **(A) *cox1* gene; (B) *cob* gene (C) *rnl* gene.** The X axis represents the gene length and the vertical lines indicate the position of introns. The numbers on top represent the relative location of each intron in different yeasts. The rectangular frames indicate Group II introns, which include introns 1, 2 and 10 in *cox1*, and intron 3 in *cob*. The triangular frames indicate the Group I introns. The filled frames indicate the introns with embedded ORFs, and the empty frames indicate introns without ORFs.

In the *SSS* group, the *cox1*, *cob* and *rnl* genes are interrupted by variable introns [24]. We found that ten intron loci in the *cox1* gene are not conserved within the *SSS* group (Fig 2A), which was consistent with a previous study of 104 *S*. *cerevisiae* strains [24]. Three group II introns (1, 2, 10) were only observed in the *SSS* genomes. Among them, the putative ORFs in intron 1 and intron 2 are highly conserved in two *SSS* yeasts (amino-acid identity of 99% and 94%, respectively, S7 Table), and intron 10, which does not contain ORFs, also has a high nucleotide identity of 83% in all *SSS* yeasts. With the exception of introns 3 and 4, all the remaining group I introns (5, 6, 7, 8, 9) contained ORFs. Among them, intron 5 was mainly distributed in the *SSS* group with a low amino-acid identity of 71%, whereas intron 8 had a high amino-acid identity in both the *Lachancea* and *SSS* linages (97% and 95%). Secondly, seven loci in the *cob* gene were found in fifteen yeasts (Fig 2B). Except for a rare intron (1) which was only observed in *K*. *servazzii*, the remaining six loci can be found in the *SSS* group. Most of the introns in the *cob* gene had lower identity scores than those in the *cox1* gene. Only intron 3, which belongs to group II, had a high degree of nucleotide identity (83%), while intron 5 had a high degree of amino-acid identity in both the *Lachancea* and *SSS* linages (95% and 99%, respectively). Lastly, the *rnl* gene only contained one intron in the *SSS*, K/N and *Lachancea* linages, but not in the C/N linage (Fig 2C). Among them, only the intron in *S*. *cerevisiae* contained an ORF.

In summary, seventeen introns were identified in the *SSS* group, much more than the six in the K/N linage, seven in the C/N linage and ten in the *Lachancea* genus. Therefore, the expansion of introns appears to have also played an important role in the expansion of MT genomes. Most intron sequences, especially of Group II introns, are highly conserved in the *SSS* group. However, the presence of introns and their embedded ORFs changed remarkably throughout the group. In addition, a total 221 out of 282 introns (~78%) in the nuclear genome of *S*. *cerevisiae* are conserved in the other four species in the *SSS* group [25]. By contrast, only one out of 12 introns (8%) in the MT genome of *S*. *cerevisiae* is conserved in the other four species from the *SSS* group. The presence of introns in MT genomes thus changed more rapidly than in nuclear genomes.

### Rapid evolution of genomic structure

**Fig 3:**
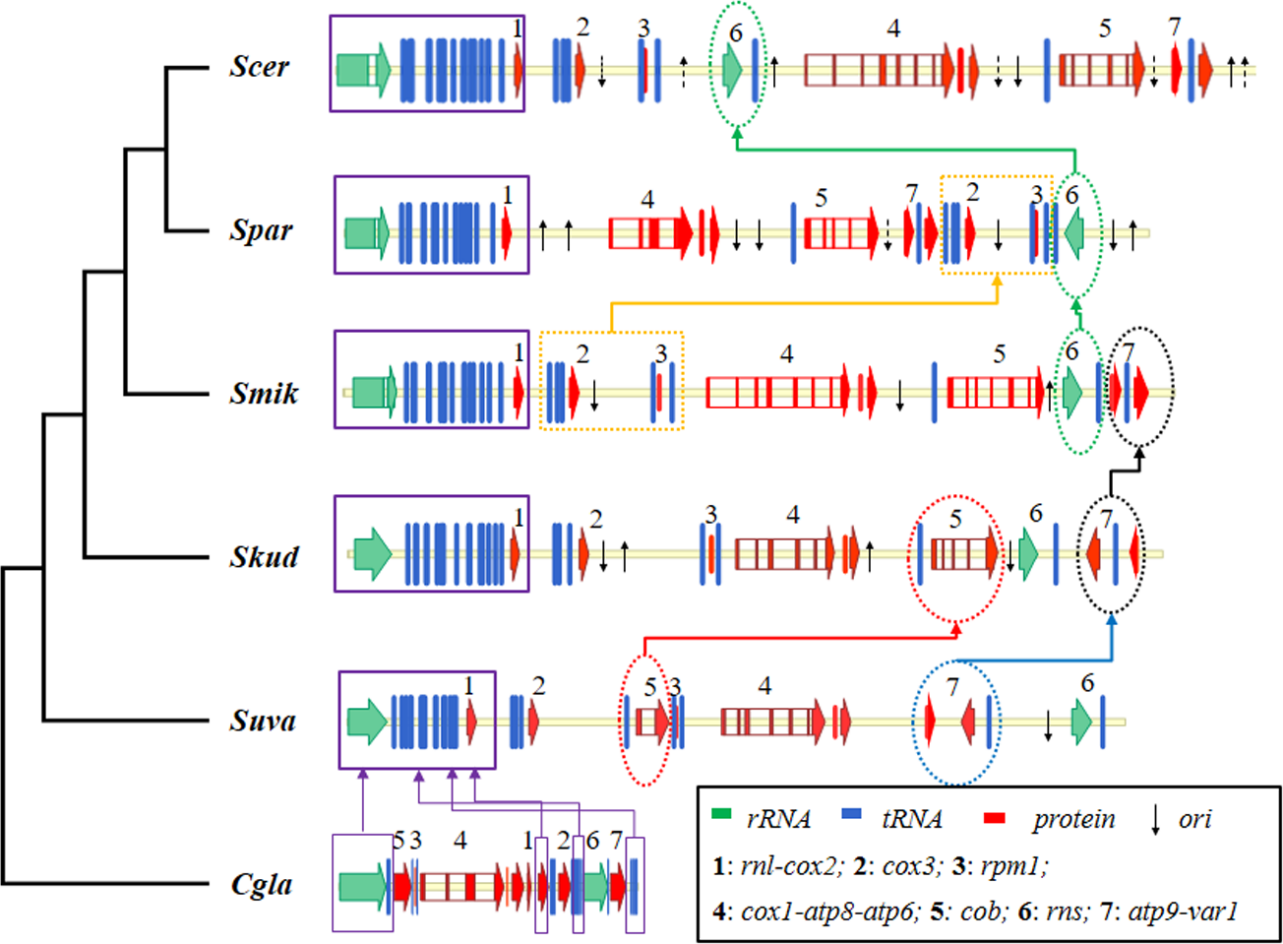
Evolution of gene order within the *Saccharomyces sensu stricto* group. Block1 includes *rnl*, *tRNAs* (T2,C,H,L,Q,K,R1,G,D,S1,R2,A,I,Y,N,M1) and *cox2*. Block2 includes *tRNAs* (F,T1,V), and *cox3*. Block3 includes *tRNA* (M2), *rpm1* and *tRNA* (P). Block4 includes *cox1*, *atp8* and *atp6*. Block5 includes *tRNA* (E) and *cob*. Block6 includes *rns* and *tRNA* (W). Block7 includes *atp9*, *tRNA* (S2) and *var1*. The downward and upward black arrows indicate the *ori* sequences in the positive and negative strands, respectively. The dashed arrows indicate that the *ori* sequences contain intervening GC clusters.

There is high variance in the order of mitochondrial genes among fungi [26]. In comparison with the conserved gene order in the genus *Lachancea* [13], the gene order displayed considerable diversity in the *SSS* group [19]. To investigate the evolution of gene order in the *SSS* group, we divided all 35 genes into seven syntenic orthologous blocks, consisting of fourteen known transcriptional units in *S*. *cerevisiae* (S8 Table and Fig 3). In total, we detected 25 rearrangement events, including fourteen transpositions, ten inversions and one inverse transposition across the *SSS* group, by pairwise comparisons using CREx [27] and UniMoG [28] (S9 Table). Details of the rearrangement scenarios are shown in S1B File. Except for block 7 (*atp9* and *var1-trnS2*), which involved an inner inverse transposition in *S*. *uvarum*, the other rearrangement events were block transpositions or inversions. We also found that all of the *ori* sequences were distributed upstream or downstream of the blocks (Fig 3), and especially, the rearrangement hotspot of block 6 (*rns* and *trnW*) had an upstream *ori* in all *SSS* yeasts. Therefore, we inferred that the *ori* sequences may play an important role in gene rearrangements in the *SSS* group.

We further employed the rate of branch-specific gene order loss (bsGOL) to measure the rearrangement rate by methods developed in previous studies [29, 30] (S10 Table). We found that the rearrangement rate in the *SSS* group was faster than in other lineages. The gene order in the *Lachancea* lineage was the most stable (the bsGOL rate was almost 0, except for *L*. *kluyveri*), followed by the *K/N* lineage, and then by *C*. *glabrata* and *N*. *delphensis* in the *C/N* lineage (S10 Table). Overall, *S*. *paradoxus* had the fastest rearrangement rate, which was about 20 times faster than the rates in the nuclear genomes of *S*. *cerevisiae* and *S*. *paradoxus* [30]. The rapid evolution of mitochondrial gene order in this species might be associated with a high abundance of *ori* sequences in the mtDNA.

### High conservation of functional genes in MT genomes

**Fig 4:**
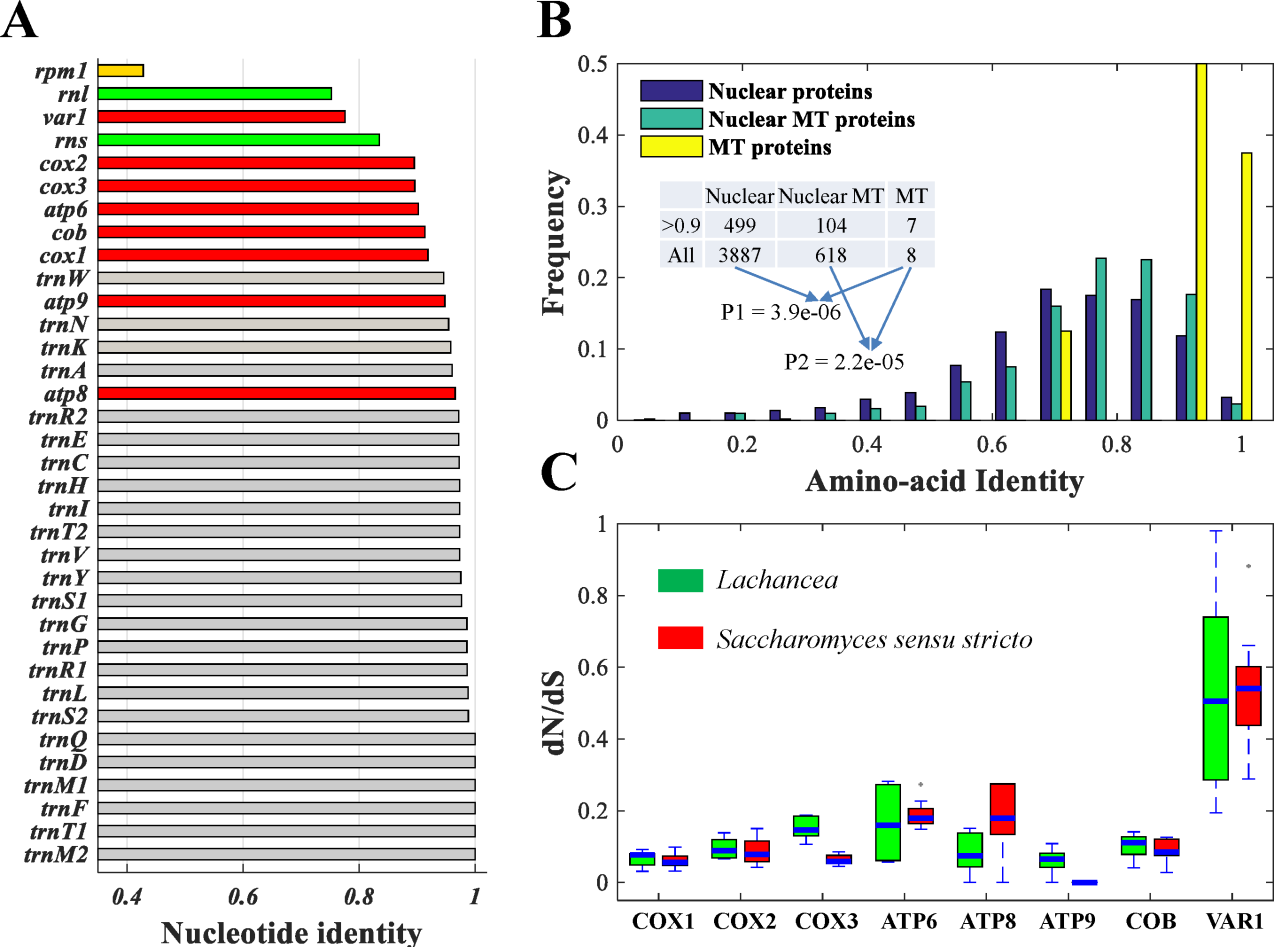
(A) The nucleotide identities of all mitochondrial genes in the *Saccharomyces sensu stricto* group. The nucleotide identity was calculated based on the proportion of completely conserved nucleotides in multiple sequence alignments of five *SSS* yeasts conducted using ClustalOmega. The red bars represent protein-coding genes; the green bar is rRNA; the gray bar is tRNA and the yellow bar is *rpm1*. (B) Comparison of amino-acid identities among nuclear proteins, nuclear MT proteins and mitochondrial proteins. We identified 3,887 nuclear proteins which are present in all five *SSS* yeasts. Among them, 618 proteins were located in the mitochondria. The amino-acid identity for each protein was calculated based on the proportion of completely conserved amino-acid residues in the multiple sequences alignment (MSA) results. The dark blue, green and yellow bars represent the distribution of nuclear proteins, nuclear MT proteins and mitochondrial proteins, respectively. The table insert indicates the number of proteins which have identities greater than 90% in three protein sets. The *p*-values were calculated based on a hypergeometric test whether the number of MT proteins with high identity was significantly greater than those in the other two protein sets. (C) Box-plot comparisons of the dN/dS ratios estimated for the eight MT protein-coding genes between the genus *Lachancea* and the *Saccharomyces sensu stricto* linage.

In contrast to the dramatic expansion of intergenic regions and rapid evolution of gene order in the *SSS* group, most functional genes are highly conserved in MT genomes (Fig 4A)[31]. For example, all 24 tRNAs have nucleotide identities >95% based on multiple sequence alignment within the *SSS* group. The same pattern is also observed in nuclear tRNAs (S11 Table)[32]. Two rRNAs (*rnl* and *rns*), and the *rpm1* gene have lower identities compared to other MT genes and the corresponding RNAs in nuclear genomes (S11 Table). Except for *var1*, which has ~80% nucleotide identity, the other seven protein-coding genes are highly conserved (with identities > 90%). We also found that seven proteins have significantly higher amino-acid identities than the functional proteins in the nuclear genomes or nuclear proteins with mitochondrial functions (Fig 4B)[33]. We furthermore inferred natural selection on these proteins by estimating the ratio of non-synonymous (dN) to synonymous (dS) substitution rates (ω = dN/dS) (Fig 4C and S12 Table). In the *Saccharomyces* linages, all of the proteins were under purifying selection (ω < 1). The *SSS* group showed stronger purifying selection in *cox3* (*t*-test, *p* = 1.7e-6) and *atp9* (*t*-test, *p* = 0.00011) than those in the *Lachancea* linage [13]. Especially in *atp9*, no non-synonymous substitutions (dN = 0) were found in the *SSS* group.

## Discussion

By comparing the MT genomes of the *SSS* group with their close relatives, we found that there was an expansion of the MT genome size. The expansion was mainly driven by divergence in intergenic regions [6]. Our results also showed that the relative number of GC clusters in intergenic regions accounted for 63% of the genome size variation (S1D Fig).

In contrast to the higher variability of intergenic and intron regions in MT genomes than in nuclear genomes, coding regions are more conserved in MT genomes than in nuclear genomes of the *SSS* group, which indicates that the mitochondrial genes underwent stronger purifying selection [34, 35]. It is likely that the retained genes in the MT genomes are indispensable for cell respiration [36] and the high AT content of MT genomes (approximately 80%) restricted mutations of mitochondrial genes [37, 38]. Moreover, we speculated that the origin of the aerobic fermentation lifestyle in the *SSS* group could also be associated with the opposite evolutionary process for coding regions and non-coding regions between MT genomes and nuclear genomes. In rich glucose media, yeast cells can rapidly grow via fermentation without functional mitochondria, and thus mitochondrial genomes possibly could evolve rapidly. However, when the sugar is exhausted, the mitochondria become indispensable for energy generation, and thus the functional mitochondrial genes are subject to very strong selection.

## Materials and methods

### Strains and DNA preparation

*S*. *mikitae* strain IFO1815 and *S*. *kudriavzevii* strain IFO1802 used in this study were from Cornell University. Yeast cells were grown in YPD medium (2% glucose, 1% yeast extract, and 2% peptone) at 30°C with 220 rpm overnight. Then 10ml of cell culture was transferred into 800ml of fresh YPG liquid medium (3% glycerol, 1% yeast extract and 2% peptone) [39]. The entire mitochondrial genomic DNAs of *S*. *mikatae* and *S*. *kudriavzevii* were purified by standard protocol [40].

### Sequencing and assembly

The entire *S*. *mikatae* mtDNA was sequenced by Illumina sequencing platforms and assembled by SOAPdenovo pipeline [41]. The *S*. *kudriavzevii* mtDNA was mainly obtained from the whole genome sequencing of *S*. *kudriavzevii* [21], which was downloaded from SGD (http://downloads.yeastgenome.org/sequence/fungi/S_kudriavzevii/IFO1802/). We first assembled five contigs (~71kb) for *S*. *kudriavzevii* mtDNA by comparing with the referential mtDNA of *S*. *cerevisiae*. Then, the gaps between contigs were filled by PCR. Finally, we combined assembled PCR results with contigs to get the complete sequence of *S*. *kudriavzevii* mtDNA. The complete sequences of *S*. *mikatae* and *S*. *kudriavzevii* mtDNA had been deposited at NCBI under the accessions KX707787 and KX707788.

### Gene annotation

The mitochondrial genomes of *S*. *mikatae* and *S*. *kudriazevii* were annotated based on all mitochondrial genes in *S*. *cerevisiae* [19]. The location of tRNAs, rRNAs, and introns were detected by tRNAscan [42], RNAweasel [43] and MFannot [44].

### mtDNA sequences

Yeast mtDNA sequences were obtained from NCBI and EMBL. The accession numbers are AJ511533 (*Candida glabrata*), CM003593 (*S*. *uvarum*), KR260476 (*S*. *cerevisiae*), JQ862335 (*S*. *paradoxus*), AJ430679 (*Kazachstania servazzii*), AF437291 (*Naumovozyma castellii*), FM995164 (*Nakaseomyces delphensis*), FM995165 (*Candida castellii*), FM995166 (*Nakaseomyces bacillisporus*), HE664111 (*Lachancea kluyveri*), AJ634268 (*Lachancea thermotolerans*), HE983611 (*Lachancea dasiensis*), and HE983614 (*Lachancea meyersii*).

### The organization architecture of the mitochondrial genomes

To investigate the organization and evolution of the mitochondrial genome in the *SSS* group, we classified the mitochondrial genome into five parts: the first was gene sequences, including 35 genes (8 proteins, 2 rRNAs, 1 RNase P and 24 tRNAs) for all of *SSS* group; the second was intron sequences, including 7–14 introns distributed in *cox1*, *cob* and *rnl*; the third was intergenic regions, consisting of 33–35 intergenic sequences within each *SSS* yeast; the fourth was *ori* sequences, including 1–8 *ori* sequences distributed in the intergenic regions; and the last was *ORF* sequences located between (or at) the intergenic regions.

### Constructing phylogenetic tree

The amino acid sequences of eight protein coding genes in the fifteen yeasts were aligned by ClustalOmega [31] separately, trimmed by trimAl (gap-score cutoff 0.5, conservation score 0.5) [45], and then concatenated into a single alignment. Then, a maximum-likelihood tree was constructed by MEGA [46]. All nodes were highly supported by the likelihood ratio test and bootstrap analysis with 100 replications.

### Constructing bsGOL rate

Gene order conservation (GOC) was defined as the number of contiguous orthologous pair in two genomes (N_orthologues, contiguous_) divided by the total number of orthologues (N_orthologues_) [29]. All the GOC values between the 15 yeasts were estimated in phylogenetic tree (Fig 1C) as:

$$GOC=\frac{N_{orthologues,contiguous}}{N_{orthologues}}$$

The gene order loss (GOL) was defined as 1-GOC. Branch-specific GOL (bsGOL) could be obtained by minimizing the sum over 105 pairwise comparisons, of the squared differences between the frequency of the observed GOL and the sum of the predicted bsGOL values, the likelihood function is:

$$L=\sum_{i=1}^{105}{\left(\sum_{j=1}^{27}b_{i,j}x_{j}-{GOL}_{i}\right)}^{2}$$

where *b*_*i*,*j*_ was a Boolean variable indicating the specific relation for the estimation of a particular bsGOL, GOL_*i*_ were obtained from the pairwise comparisons (GOL_*i*_ = 1-GOC_i_), *x*_*j*_ was the actual bsGOL (S10 Table) value apply for minimizing **L** [30].

### Calculating dN/dS ratios

The method for calculating dN/dS ratios was from a previous study [13]. Firstly, the phylogenetic tree based on the concatenation of eight protein coding genes was used as reference tree for estimating dN/dS in PAML package version 4.4b [47]. Secondly, the nucleotide sequence of each protein coding gene was translated to amino acid sequence for multiple sequences alignment (MSA) by ClustalOmega [31], and then the MSA of each nucleotide sequence was produced by MSA amino acid sequence. Lastly, CODEML model was used to estimate the pairwise dN/dS ratios among all linages in our study.

### Predicting GC clusters and AT spacers

The GC cluster was first detected with the minimum length 16bp and minimum GC content 0.75. Then, we masked the mtDNA with the detected GC cluster sequences and further detected new GC clusters in the remaining mtDNA sequences with the minimum length 8bp and minimum GC content 0.85. And last, two clusters were joined together if the gap was less than 10bp.

To group the GC clusters in one species, we first conducted a multiple sequence alignment for all GC clusters and their reverse compliment sequences by ClustalOmega. Secondly, we constructed the evolutionary tree based on multiple sequence alignments, and divided GC clusters into different groups based on the tree with a threshold. Thirdly, we filtered the reverse compliment sequence with a GC cluster, if the GC cluster and its reverse compliment sequence existed in the same group. Fourthly, we sorted the groups in descending order of the number in the group, and the groups were successively retained, but some groups was removed if they had an over 80% overlap with the retained groups. And lastly, we aligned the multiple sequence for all groups, and obtained the consensus sequence for each group.

To further detect the intergenic AT spacers in MT genomes, we detected the GC clusters comprising of at least one G/C tetranucleotide (e.g, GCCG, CCGC etc.). After excluding all GC clusters in the intergenic region, the remaining intergenic fragments were considered as AT spacers, if the sequence size >20bp and GC content < 0.1.

### Determining intron position

If an intron (list in Fig 2) located at the correct position in the gene sequences, we used two criteria: 1), the introns located at the neighboring positions in different species, and 2), conservation of the 5' and 3' flanking exon sequences around the same intron loci in different species. The latter criteria was more important and the flanking sequences around 5' and 3' region (10bp) for all 18 introns were listed in S6 Table.

## Supporting information

S1 Fig (A) Scatter plot and line regression of the relationship between the size of MT genome and intergenic regions. (B) The expansion of intergenic region of mtDNA. The intergenic sequences were divided into four types: ORF sequences, *ori* sequences, AT spacers and GC clusters. The Y axis represented the size of four types of intergenic regions, for detailed data, see the S3 Table. (C) The (AT+TA)/(AA+TT) ratio in the AT spacers of fifteen yeasts. (D) Scatter plot and line regression of the relationship between the MT genome size and the relative number of GC clusters. The scatter plots in red ellipse represented five *SSS* yeasts.
(PDF)

S2 Fig The distribution of GC clusters in mitochondrial genomes. The gray color referred to no GC cluster insertion; the blue color referred to fewer GC clusters insertion (1–3); the red color referred to more GC clusters insertion (8–10); the dark red referred to the number of GC clusters is larger than 10. The detailed number of GC clusters was shown in S4 Table.
(PDF)

S3 Fig The logos Figure of *ori* sequences for five *SSS* yeasts. All 24 *ori* sequences were aligned with MEGA software [46]. The intervening GC clusters and AT segment in some *ori* sequences were removed before drawing. The Figure was created by WebLogo[23].
(PDF)

S4 Fig The number symbol for each evolutionary branch in the phylogenetic tree. The tree had totally 27 evolutionary branches.
(PDF)

S1 Table Gene coordinates in mitochondrial genomes of *S*. *mikatae* and *S*. *kudriavzevii*. The table showed the coordinates of 35 genes and their introns in *S*. *mikatae* and *S*. *kudriavzevii*.
(PDF)

S2 Table The MT genome sizes of fifteen yeasts. The size of genes, introns and intergenic regions in the mtDNA of *L*. *meyersii (Lmey)*, *L*. *dasiensis (Ldas)*, *L*. *thermotolerans (Lthe)*, *L*. *kluyveri (Lklu)*, *N*. *bacillisporus (Nbac)*, *C*. *castellii (Ccas)*, *N*. *delphensis (Ndel)*, *C*. *glabrata (Cgla)*, *N*. *castellii (Ncas)*, *K*. *servazzii (Kser)*, *S*. *uvarum (Suva)*, *S*. *kudriavzevii (Skud)*, *S*. *mikatae (Smik)*, *S*. *paradoxus (Spar)*, and *S*. *cerevisiae (Scer)*.
(PDF)

S3 Table The size of *ori* sequences, ORFs, AT spacers and GC clusters in intergenic region. The number in the bracket of ‘ORFs’ and ‘Oris’ cols indicated the number of ORFs and *ori* sequences in different yeasts.
(PDF)

S4 Table The number of GC clusters in fifteen yeasts. The mitochondrial genomes were divided into ‘Exon’, ‘Introns’ and ‘Intergenic’ regions. The ‘Sum’ col indicated sum of GC clusters in different yeasts.
(PDF)

S5 Table The distribution and characteristic of GC clusters in different yeast linages. The ‘Num’ col indicated the number of GC clusters in the MT genome of each yeast. The ‘Palindromic’ col indicated the proportion of palindromic-like GC clusters. The ‘FamilyNum’ indicated the number of the main sub-families in each yeast (number > = 8). The ‘Consensus’ and ‘RC Consensus’ cols indicated the consensus sequence and the reverse complementary sequence of each subfamily. In the ‘Features’ col, * = The al and a2 family in de Zamaroczy and Bernardi, 1986; ^&^ = GC clusters A, B, C of *ori* sequences.
(PDF)

S6 Table The 10 bp 5' and 3' flanking exon sequences of introns. The number after a series of ‘#’ represented the relative location of each intron in different yeasts. The flanking sequence was named according to ‘Species_Gene_ID_Loc’, where the ‘ID’ indicated the absolute location of the intron in corresponding gene and species, the ‘Loc’ indicated the insert location of the intron in the coding sequence. For example, ‘Smik_COX1_1_168’ represented the first intron of *cox1* gene in MT genome of *S*. *mikatae*, the intron inserted in the 168th nucleotide of coding sequence. The flanking sequence was shown according to “5’ 10 bp exon sequence-3’ 10 bp exon sequence”, where the ‘-’ represented the insert location of the intron in the coding sequence.
(PDF)

S7 Table The intron identity in four yeast linages. We first calculated the amino-acid identity of intron ORFs in different linages. We then calculated the nucleotide identity for the introns without ORF. The rows with yellow back color were corresponding to Group II introns. The ‘-’ indicated the intron did not present in the linage, or only present in one species of the linage.
(PDF)

S8 Table Seven blocks in mitochondria genome of *SSS* group. All 35 genes were divided into seven syntenic orthologous blocks constituted by fourteen transcriptional units known in *S*. *cerevisiae*. For other *SSS* yeasts, we predicted transcription initiation site based on the motif WTATAAGTA. The predicted transcriptional units were similar to that in *S*. *cerevisiae* (data not shown).
(PDF)

S9 Table The number of rearrangement events between *SSS* yeasts. The upper triangle of the table indicated the number of rearrangement events. The rearrangement scenarios were shown in supplement file (S1B File) in more detail.
(PDF)

S10 Table Branch-Specific Rates of GOL in fifteen yeasts. The ‘Branch’ col indicated all evolutional branches in the phylogenetic tree of fifteen yeasts (S4 Fig). The ‘Leaf_Node’ col indicated the branches which included leaf nodes (i.e., fifteen yeasts). The ‘Branch Length’ col indicated the length of each branch in the phylogenetic tree. The ‘MT bsGOL’ indicated the branch-specific GOL of mitochondrial genome based on the pairwise comparisons of GOL. The ‘MT GOL Rate’ indicated the rearrangement rate of gene order in mitochondrial genome which was calculated by the ratio of bsGOL to branch length. The ‘Nuclear GOL Rate’ indicated the GOL rates in the nuclear genome of *S*. *cerevisiae* and *S*. *paradoxus* which were from *Fischer et al*. 2006 [30].
(PDF)

S11 Table The identity between nucleus and mitochondrial RNA genes. We first predicted all nucleus tRNAs, rRNAs and RPR1 in other *SSS* yeasts based on the genes annotation in *S*. *cerevisiae*. Then we obtained the orthologous groups for all RNA genes by OrthoMCL [32]. After that, we calculated the nucleotide identity for each orthologous group, if the group included genes in all five *SSS* yeasts.
(PDF)

S12 Table The dN/dS ratio for all linages. The ‘Average’ rows in the table indicated the average of the dN/dS ratio of each mitochondria protein between two close relative species in the same lineage. The number in ‘Rank’ rows represent the rank number based on the ‘Average’.
(PDF)

S1 File (A) Intergenic regions expansion. (B) The rearrangement scenarios in the *SSS*.
(PDF)
