# Supplementary material for: Mitochondrial genome evolution in the Saccharomyces sensu stricto complex
Source: PLoS One. 2017 Aug 16;12(8):e0183035. doi: 10.1371/journal.pone.0183035 (PMC5558958; doi:10.1371/journal.pone.0183035)
Supplement: S1 Fig — (A) Scatter plot and line regression of the relationship between the size of MT genome and intergenic regions. (B) The expansion of intergenic region of mtDNA. The intergenic sequences were divided into four types: ORF sequences, ori sequences, AT spacers and GC clusters. The Y axis represented the size of four types of intergenic regions, for detailed data, see the S3 Table. (C) The (AT+TA)/(AA+TT) ratio in the AT spacers of fifteen yeasts. (D) Scatter plot and line regression of the relationship between the MT genome size and the relative number of GC clusters. The scatter plots in red ellipse represented five SSS yeasts. (PDF) [file pone.0183035.s001.pdf]

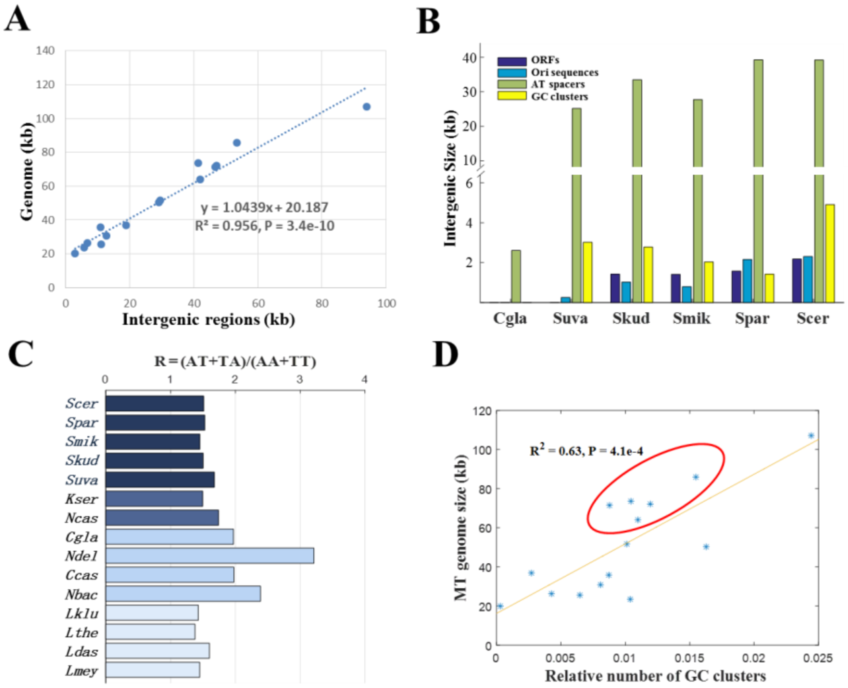

S1 Fig. (A) Scatter plot and line regression of the relationship between the size of MT genome and intergenic regions. (**B) The expansion of intergenic region of mtDNA.** The intergenic sequences were divided into four types: ORF sequences, *ori* sequences, AT spacers and GC clusters. The Y axis represented the size of four types of intergenic regions, for detailed data, see the S3 Table. **(C) The (AT+TA)/(AA+TT) ratio in the AT spacers of fifteen yeasts.** (**D) Scatter plot and line regression of the relationship between the MT genome size and the relative number of GC clusters.** The scatter plots in red ellipse represented five *SSS* yeasts.
