# Supplementary material for: Mitochondrial genome evolution in the Saccharomyces sensu stricto complex
Source: PLoS One. 2017 Aug 16;12(8):e0183035. doi: 10.1371/journal.pone.0183035 (PMC5558958; doi:10.1371/journal.pone.0183035)
Supplement: S2 Fig — The gray color referred to no GC cluster insertion; the blue color referred to fewer GC clusters insertion (1–3); the red color referred to more GC clusters insertion (8–10); the dark red referred to the number of GC clusters is larger than 10. The detailed number of GC clusters was shown in S4 Table. (PDF) [file pone.0183035.s002.pdf]

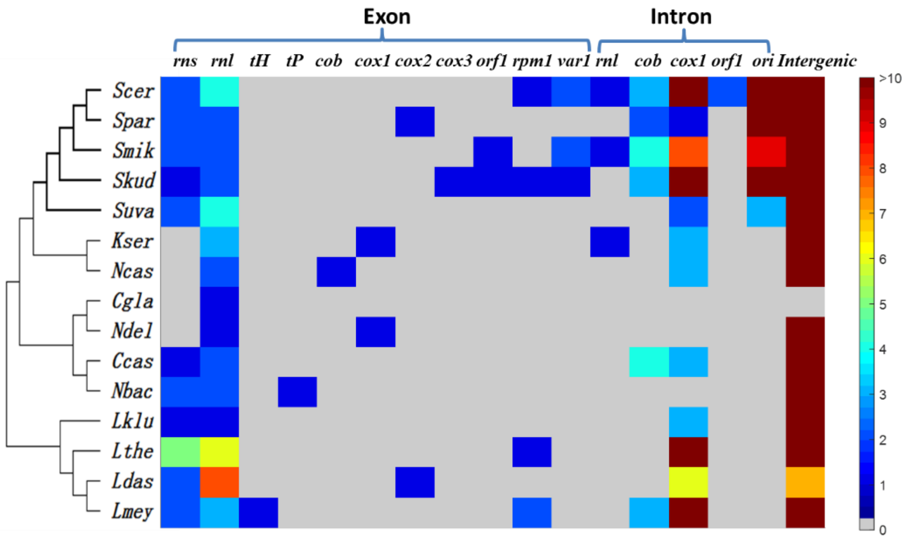

S2 Fig. The distribution of GC clusters in mitochondrial genomes. The gray color referred to no GC cluster insertion; the blue color referred to fewer GC clusters insertion (1 - 3); the red color referred to more GC clusters insertion (8 - 10); the dark red referred to the number of GC clusters is larger than 10. The detailed number of GC clusters was shown in S4 Table.
