# Supplementary material for: Mitochondrial genome evolution in the Saccharomyces sensu stricto complex
Source: PLoS One. 2017 Aug 16;12(8):e0183035. doi: 10.1371/journal.pone.0183035 (PMC5558958; doi:10.1371/journal.pone.0183035)
Supplement: S3 Fig — All 24 ori sequences were aligned with MEGA software [46]. The intervening GC clusters and AT segment in some ori sequences were removed before drawing. The Figure was created by WebLogo[23]. (PDF) [file pone.0183035.s003.pdf]

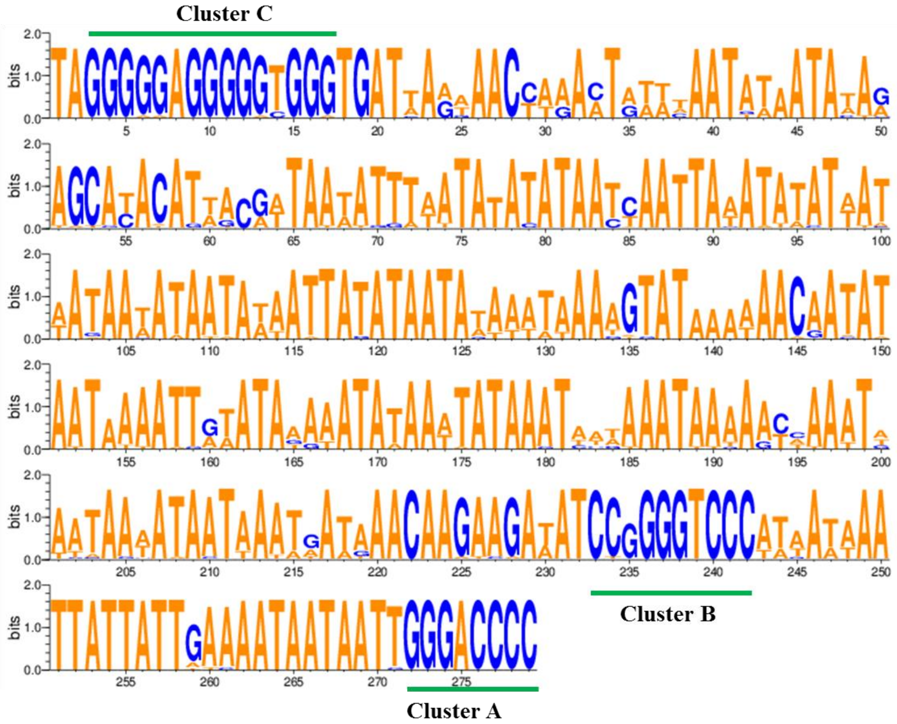


**S3 Fig. The logos figure of *ori* sequences for five *SSS* yeasts**. All 24 *ori* sequences were aligned with MEGA software (Tamura, Stecher et al. 2013). The intervening GC clusters and AT segment in some *ori* sequences were removed before drawing. The figure was created by WebLogo (Crooks, Hon et al. 2004).
