# Supplementary material for: Mitochondrial genome evolution in the Saccharomyces sensu stricto complex
Source: PLoS One. 2017 Aug 16;12(8):e0183035. doi: 10.1371/journal.pone.0183035 (PMC5558958; doi:10.1371/journal.pone.0183035)
Supplement: S4 Fig — The tree had totally 27 evolutionary branches. (PDF) [file pone.0183035.s004.pdf]

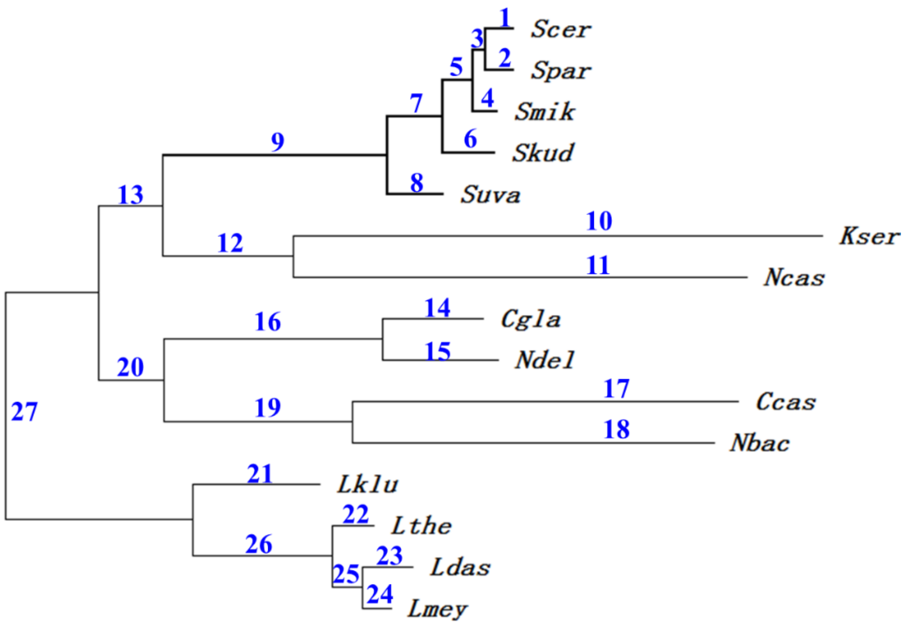

S4 Fig. The number symbol for each evolutionary branch in the phylogenetic tree. The tree had totally 27 evolutionary branches.
