# Supplementary material for: Mitochondrial genome evolution in the Saccharomyces sensu stricto complex
Source: PLoS One. 2017 Aug 16;12(8):e0183035. doi: 10.1371/journal.pone.0183035 (PMC5558958; doi:10.1371/journal.pone.0183035)
Supplement: S1 Table — The table showed the coordinates of 35 genes and their introns in S. mikatae and S. kudriavzevii. (PDF) [file pone.0183035.s005.pdf]

S1 Table. Gene coordinates in mitochondrial genomes of *S.mikatae* and *S.kudriavzevii*

S.mikatae	Coordinates			***S.kudriavzevii***	Coordinates		
Genetic element	Start	End	Orientation	*Genetic element*	Start	End	Orientation
rnl exon1	789	3469	Direct	*rnl*	537	3802	Direct
Intron rnl	3470	3862	Direct	*tRNA-Thr2*	5237	5312	Direct
rnl exon2	3863	4441	Direct	*tRNA-Cys*	5915	5987	Direct
tRNA-Thr2	5483	5558	Direct	*tRNA-His*	6025	6099	Direct
tRNA-Cys	6057	6129	Direct	*tRNA-Leu*	6849	6933	Direct
tRNA-His	6221	6295	Direct	*tRNA-Gln*	6952	7027	Direct
tRNA-Leu	7420	7504	Direct	*tRNA-Lys*	7817	7888	Direct
tRNA-Gln	7528	7603	Direct	*tRNA-Arg1*	8190	8262	Direct
tRNA-Lys	8507	8580	Direct	*tRNA-Gly*	8400	8471	Direct
tRNA-Arg1	8733	8805	Direct	*tRNA-Asp*	9560	9632	Direct
tRNA-Gly	8853	8927	Direct	*tRNA-Ser1*	10633	10718	Direct
tRNA-Asp	9538	9612	Direct	*tRNA-Arg2*	10725	10797	Direct
tRNA-Ser1	10565	10650	Direct	*tRNA-Ala*	11650	11725	Direct
tRNA-Arg2	10651	10724	Direct	*tRNA-Ile*	11848	11922	Direct
tRNA-Ala	11109	11184	Direct	*tRNA-Tyr*	12402	12485	Direct
tRNA-Ile	11409	11484	Direct	*tRNA-Asn*	13001	13071	Direct
tRNA-Tyr	12172	12253	Direct	*tRNA-Met1*	13540	13614	Direct
tRNA-Asn	12813	12883	Direct	*cox2*	14358	15113	Direct
tRNA-Met1	13843	13918	Direct	*ORF1*	15095	16519	Direct
cox2	15048	15803	Direct	*tRNA-Phe*	18276	18347	Direct
ORF1	15785	17200	Direct	*tRNA-Thr1*	18643	18714	Reverse
tRNA-Phe	18161	18235	Direct	*tRNA-Val*	19579	19654	Direct
tRNA-Thr1	18844	18917	Reverse	*cox3*	20450	21259	Direct
tRNA-Val	19251	19326	Direct	*Rep-origin1*	24351	24594	Direct
cox3	19933	20742	Direct	*Rep-origin2*	28537	28796	Reverse
Rep-origin1	24731	24995	Direct	*tRNA-Met2*	31334	31411	Direct
tRNA-Met2	27327	27404	Direct	*rmp1*	32129	32549	Direct
rmp1	27866	28239	Direct	*tRNA-Pro*	32743	32814	Direct
tRNA-Pro	29004	29075	Direct	*cox1 exon1*	34268	34507	Direct
cox1 exon1	32094	32262	Direct	*Intron cox1.1*	34508	36071	Direct
Intron cox1.1	32263	34710	Direct	*cox1 exon2*	36072	36217	Direct
cox1 exon2	34711	34781	Direct	*Intron cox1.2*	36218	37231	Direct
Intron cox1.2	34782	36398	Direct	*cox1 exon3*	37232	37554	Direct
cox1 exon3	36399	36544	Direct	*Intron cox1.3*	37555	39633	Direct
Intron cox1.3	36545	37428	Direct	*cox1 exon4*	39634	39895	Direct
cox1 exon4	37429	37751	Direct	*Intron cox1.4*	39896	41263	Direct
Intron cox1.4	37752	39773	Direct	*cox1 exon5*	41264	41424	Direct
cox1 exon5	39774	40035	Direct	*Intron cox1.5*	41425	42297	Direct
Intron cox1.5	40036	41406	Direct	*cox1 exon6*	42298	42770	Direct
cox1 exon6	41407	41542	Direct	*atp8*	43984	44130	Direct
Intron cox1.6	41543	43088	Direct	*atp6*	44425	45204	Direct
cox1 exon7	43089	43113	Direct	*Rep-origin3*	49795	50056	Reverse
Intron cox1.7	43114	44003	Direct	*tRNA-Glu*	50623	50694	Direct
cox1 exon8	44004	44476	Direct	*cob exon1*	51650	52042	Direct
atp8	45692	45838	Direct	*Intron cob1*	52043	52685	Direct
atp6	46297	47076	Direct	*cob exon2*	52686	52707	Direct

Rep-origin2	48694	48957	Direct	*Intron cob2*	52708	53570	Direct
tRNA-Glu	52220	52291	Direct	*cob exon3*	53571	53584	Direct
cob exon1	53399	53791	Direct	*Intron cob3*	53585	54853	Direct
Intron cob1	53792	55143	Direct	*cob exon4*	54854	54930	Direct
cob exon2	55144	55165	Direct	*Intron cob4*	54931	56499	Direct
Intron cob2	55166	55947	Direct	*cob exon5*	56500	57151	Direct
cob exon3	55948	55961	Direct	*Rep-origin4*	58193	58454	Direct
Intron cob3	55962	57230	Direct	*rns*	59421	61024	Direct
cob exon4	57231	57307	Direct	*tRNA-Trp*	62635	62708	Direct
Intron cob4	57308	58869	Direct	*var1*	65351	66518	Reverse
cob exon5	58870	59119	Direct	*tRNA-Ser2*	67920	68008	Reverse
Intron cob5	59120	60607	Direct	*atp9*	69746	69976	Reverse
cob exon6	60608	60657	Direct				
Intron cob6	60658	61261	Direct				
cob exon7	61262	61613	Direct				
Rep-origin3	62514	62779	Reverse				
rns	63621	65268	Direct				
tRNA-Trp	66749	66822	Direct				
atp9	67942	68172	Direct				
tRNA-Ser2	69257	69346	Reverse				
var1	69915	71134	Reverse				

Note: The table showed the coordinates of 35 genes and their introns in *S.mikatae* and *S.kudriavzevii*.
