# Supplementary material for: Mitochondrial genome evolution in the Saccharomyces sensu stricto complex
Source: PLoS One. 2017 Aug 16;12(8):e0183035. doi: 10.1371/journal.pone.0183035 (PMC5558958; doi:10.1371/journal.pone.0183035)
Supplement: S2 Table — The size of genes, introns and intergenic regions in the mtDNA of L. meyersii (Lmey), L. dasiensis (Ldas), L. thermotolerans (Lthe), L. kluyveri (Lklu), N. bacillisporus (Nbac), C. castellii (Ccas), N. delphensis (Ndel), C. glabrata (Cgla), N. castellii (Ncas), K. servazzii (Kser), S. uvarum (Suva), S. kudriavzevii (Skud), S. mikatae (Smik), S. paradoxus (Spar), and S. cerevisiae (Scer). (PDF) [file pone.0183035.s006.pdf]

S2 Table. The MT genome sizes of fifteen yeasts

Species	All	Gene	Intron	Intergenic
Lmey	35864	13793(38%)	11157(31%)	10914(30%)
Ldas	26326	13940(53%)	5494(21%)	6892(26%)
Lthe	23584	13557(57%)	4326(18%)	5701(24%)
Lklu	51679	13768(27%)	8399(16%)	29512(57%)
Nbac	107123	13114(12%)	0(0%)	94009(88%)
Ccas	50294	13192(26%)	7931(16%)	29171(58%)
Ndel	37016	12971(35%)	5249(14%)	18796(51%)
Cgla	20063	13120(65%)	3989(20%)	2954(15%)
Ncas	25753	13027(51%)	1610(6%)	11116(43%)
Kser	30782	12781(42%)	5176(17%)	12825(42%)
Suva	63999	13565(21%)	8435(13%)	41999(66%)
Skud	72095	13766(19%)	11242(16%)	47087(65%)
Smik	73485	13823(19%)	18228(25%)	41434(56%)
Spar	71355	13531(19%)	11091(16%)	46733(65%)
Scer	85779	13951(16%)	18443(22%)	53385(62%)

Note: The size of genes, introns and intergenic regions in the mtDNA of *L. meyersii (Lmey), L. dasiensis (Ldas), L. thermotolerans (Lthe), L. kluyveri (Lklu), N. bacillisporus (Nbac), C. castellii (Ccas), N. delphensis (Ndel), C. glabrata (Cgla), N. castellii (Ncas), K. servazzii (Kser), S. uvarum (Suva), S. kudriavzevii (Skud), S. mikatae (Smik), S. paradoxus (Spar),* and *S. cerevisiae (Scer).*
