# Supplementary material for: Mitochondrial genome evolution in the Saccharomyces sensu stricto complex
Source: PLoS One. 2017 Aug 16;12(8):e0183035. doi: 10.1371/journal.pone.0183035 (PMC5558958; doi:10.1371/journal.pone.0183035)
Supplement: S3 Table — The number in the bracket of ‘ORFs’ and ‘Oris’ cols indicated the number of ORFs and ori sequences in different yeasts. (PDF) [file pone.0183035.s007.pdf]

S3 Table. The size of *ori sequences*, ORFs, AT spacers and GC clusters in intergenic region

Species	ORFs	Oris	AT spacers	GC clusters
Cgla	0	0	2610	0
Sbay	0	259(1)	25148	3023
Skud	1425(1)	1028(4)	33497	2781
Smik	1416(1)	795(3)	27733	2039
Spar	1578(2)	2162(8)	39253	1425
Scer	2181(5)	2305(8)	39202	4909

Note: The number in the bracket of ‘ORFs’ and ‘Oris’ cols indicated the number of ORFs and *ori* sequences in different yeasts.
