# Supplementary material for: Mitochondrial genome evolution in the Saccharomyces sensu stricto complex
Source: PLoS One. 2017 Aug 16;12(8):e0183035. doi: 10.1371/journal.pone.0183035 (PMC5558958; doi:10.1371/journal.pone.0183035)
Supplement: S4 Table — The mitochondrial genomes were divided into ‘Exon’, ‘Introns’ and ‘Intergenic’ regions. The ‘Sum’ col indicated sum of GC clusters in different yeasts. (PDF) [file pone.0183035.s008.pdf]

S4 Table. The number of GC clusters in fifteen yeasts.

	Exon											Intron				Intergenic		Sum
	rns	*rnl*	*tH*	*tP*	*cob*	*cox1*	*cox2*	*cox3*	*orf1*	*rpm1*	*var1*	*rnl*	*cob*	*cox1*	*orf1*	*ori*	*other*	
Scer	2	4	0	0	0	0	0	0	0	1	2	1	3	11	2	27	173	226
Spar	2	2	0	0	0	0	1	0	0	0	0	0	2	1	0	25	55	88
Smik	2	2	0	0	0	0	0	0	1	0	2	1	4	8	0	9	94	123
Skud	1	2	0	0	0	0	0	1	1	1	1	0	3	10	0	10	108	138
Suva	2	4	0	0	0	0	0	0	0	0	0	0	0	2	0	3	112	123
Kser	0	3	0	0	0	1	0	0	0	0	0	1	0	3	0	0	48	56
Ncas	0	2	0	0	1	0	0	0	0	0	0	0	0	3	0	0	28	34
Cgla	0	1	0	0	0	0	0	0	0	0	0	0	0	0	0	0	0	1
Ndel	0	1	0	0	0	1	0	0	0	0	0	0	0	0	0	0	14	16
Ccas	1	2	0	0	0	0	0	0	0	0	0	0	4	3	0	0	93	103
Nbac	2	2	0	1	0	0	0	0	0	0	0	0	0	0	0	0	280	285
Lklu	1	1	0	0	0	0	0	0	0	0	0	0	0	3	0	0	74	79
Lthe	5	6	0	0	0	0	0	0	0	1	0	0	0	14	0	0	35	61
Ldas	2	8	0	0	0	0	1	0	0	0	0	0	0	6	0	0	7	24
Lmey	2	3	1	0	0	0	0	0	0	2	0	0	3	10	0	0	48	69

Note: The mitochondrial genomes were divided into ‘Exon’, ‘Introns’ and ‘Intergenic’ regions. The ‘Sum’ col indicated sum of GC clusters in different yeasts.
