# Supplementary material for: Mitochondrial genome evolution in the Saccharomyces sensu stricto complex
Source: PLoS One. 2017 Aug 16;12(8):e0183035. doi: 10.1371/journal.pone.0183035 (PMC5558958; doi:10.1371/journal.pone.0183035)
Supplement: S5 Table — The ‘Num’ col indicated the number of GC clusters in the MT genome of each yeast. The ‘Palindromic’ col indicated the proportion of palindromic-like GC clusters. The ‘FamilyNum’ indicated the number of the main sub-families in each yeast (number > = 8). The ‘Consensus’ and ‘RC Consensus’ cols indicated the consensus sequence and the reverse complementary sequence of each subfamily. In the ‘Features’ col, * = The al and a2 family in de Zamaroczy and Bernardi, 1986; & = GC clusters A, B, C of ori sequences. (PDF) [file pone.0183035.s009.pdf]

S5 Table. The distribution and characteristic of GC clusters in different yeast linages

Species	Num	Palindromic	FamilyNum	Consensus	RC Consensus	Features
Lmey	69	17%	25	GGGGGGGGGGGGG	CCCCCCCCCCCCC	ploy(G)s or poly(C)s
			9	GTGTGGGGGGGC	GCCCCCCCACAC	
Ldas	24	12%	21	GGAGGGGG	CCCCCTCC	
Lthe	61	28%	13	GGGGGGGGGGAGG	CCTCCCCCCCCCC	
			12	CCTTCGGGGGGGG	CCCCCCCCGAAGG	
			8	CTTGGGGGGGGGGG	CCCCCCCCCCCAAG	
			8	GGAGGGGGGGGGG	CCCCCCCCCCTCC	
Lklu	79	13%	24	GTGACCGGCGGG	CCCGCCGGTCAC	
			11	GCCACCCCCCCCC	GGGGGGGGGTGGC	
			8	CCTCCCCCCCCCCC	GGGGGGGGGGGAGG	
Nbac	285	25%	75	GACGCGGGG	CCCCGCGTC	------
			49	CCCGCGGC	GCCGCGGG	
			36	CCCGGCAC	GTGCCGGG	
			20	GGACTACGGGGGGG	CCCCCCCGTAGTCC	
Ccas	103	48%	52	GGCCCCGGG	CCCGGGGCC	
			16	GACCCCGGGGGGG	CCCCCCCGGGGTC	
			12	GGGGCTAGCCGGGG	CCCCGGCTAGCCCC	
Ndel	16	18%	17	GGTCCGGG	CCCGGACC	
Ncas	34	14%	8	GGGACGGCGGG	CCCGCCGTCCC	------
			8	GGGGGGGGGGCG	CGCCCCCCCCCC	
Kser	56	20%	9	CCGCCGGGAG	CTCCCGGCGG	
Sbay	123	31%	32	GCCCCGGGCCCCGCGGGCCCGGGACCCCGCTAGGAG	CTCCTAGCGGGGTCCCGGGCCCGCGGGGCCCGGGGC	a2 like[*]
			19	GTCGGGGGGGGCCGGACGCCGGAGGAG	CTCCTCCGGCGTCCGGCCCCCCCCGAC	------
			17	CGGGTCCC	GGGACCCG	Cluster B[&]
			13	CTCCTACGGCGTCCGCCCCCCCCGGGGGGGGC	GCCCCCCCCGGGGGGGGCGGACGCCGTAGGAG	------
Skud	138	22%	50	CCGGCCCCAGCCGGGACCCCGTTAGGG	CCCTAACGGGGTCCCGGCTGGGGCCGG	a2 like[*]
			45	CTCCTTACGGGGTCCCGGCTGGGGCCGGGAC	GTCCCGGCCCCAGCCGGGACCCCGTAAGGAG	a2 like[*]
			24	CGGGGTTCCGGCTCCCGTACCGGGCCCCGGAAC	GTTCCGGGGCCCGGTACGGGAGCCGGAACCCCG	------
			18	CCGGCCCCCCCCCCG	CGGGGGGGGGGCCGG	------
Smik	123	28%	20	CGGGGTCCC	GGGACCCCG	Cluster A[&]
			20	GTCCCGGCCCCAGCCGGGACCCCGAGGAG	CTCCTCGGGGTCCCGGCTGGGGCCGGGAC	a2 like[*]
			18	GGCCCCTAACCGGGTCCCGGTG	CACCGGGACCCGGTTAGGGGCC	------
Spar	88	30%	30	CCGGGACCCGG	CCGGGTCCCGG	Cluster B[&]
			17	GGTGGGGGTCCC	GGGACCCCCACC	Cluster A[&]
			10	GGGGGAGGGGGTGGGTG	CACCCACCCCCTCCCCC	Cluster C[&]
Scer	226	33%	47	CTCCTTTCGGGGTTCCGGCTCCCGTGGCCGGGCCCCGGAAC	GTTCCGGGGCCCGGCCACGGGAGCCGGAACCCCGAAAGGAG	a1[*]
			27	GTCCGGCCCGCCCCCGCGGGGCGGACCCCGAAGGAG	CTCCTTCGGGGTCCGCCCCGCGGGGGCGGGCCGGAC	a2[*]
			23	GGGACCCCC	GGGGGTCCC	Cluster A[&]
			18	GGGGACCCGG	CCGGGTCCCC	Cluster B[&]

Note: The ‘Num’ col indicated the number of GC clusters in the MT genome of each yeast. The ‘Palindromic’ col indicated the proportion of palindromic-like GC clusters. The ‘FamilyNum’ indicated the number of the main sub-families in each yeast (number > = 8). The ‘Consensus’ and ‘RC Consensus’ cols indicated the consensus sequence and the reverse complementary sequence of each subfamily. In the ‘Features’ col, [*] = The al and a2 family in de Zamaroczy and Bernardi, 1986; [&] = GC clusters A, B, C of *ori* sequences.
