# Supplementary material for: Mitochondrial genome evolution in the Saccharomyces sensu stricto complex
Source: PLoS One. 2017 Aug 16;12(8):e0183035. doi: 10.1371/journal.pone.0183035 (PMC5558958; doi:10.1371/journal.pone.0183035)
Supplement: S6 Table — The number after a series of ‘#’ represented the relative location of each intron in different yeasts. The flanking sequence was named according to ‘Species_Gene_ID_Loc’, where the ‘ID’ indicated the absolute location of the intron in corresponding gene and species, the ‘Loc’ indicated the insert location of the intron in the coding sequence. For example, ‘Smik_COX1_1_168’ represented the first intron of cox1 gene in MT genome of S. mikatae, the intron inserted in the 168th nucleotide of coding sequence. The flanking sequence was shown according to “5’ 10 bp exon sequence-3’ 10 bp exon sequence”, where the ‘-’ represented the insert location of the intron in the coding sequence. (PDF) [file pone.0183035.s010.pdf]

S6 Table. The 10 bp 5' and 3' flanking exon sequences of introns

The flanking sequences of 10 COX1 introns	
## 1	
Smik_COX1_1_168	TTATTTAATG-TTTTAGTAGT
Scer_COX1_1_168	TTATTTAATG-TTTTAGTAGT
## 2	
Spar_COX1_1_204	ATGATTTTCT-TCTTAGTAAT
Scer_COX1_2_203	ATGATTTTCT-TCTTAGTAAT
## 3	
Ccas_COX1_1_242	AGCTTATGGT-AATTATATGT
Ncas_COX1_1_239	AGGGTTTGGT-AATTATATGT
Ndel_COX1_1_239	AGGTTTTGGT-AACTATATGT
Cgla_COX1_1_239	AGGTTTTGGT-AACTATATGT
Kser_COX1_1_239	AGGTTTTGGT-AATTATATGT
Sbay_COX1_1_239	TGGGTTTGGT-AATTATTTAT
Smik_COX1_2_238	AGGTTTTGGT-AATTATTTAT
Lmey_COX1_1_239	AGGATTTGGT-AACTATTTAT
Ldas_COX1_1_239	AGGATTTGGT-AACTATTTAT
Lthe_COX1_1_239	AGGGTTTGGT-AACTATTTAT
Lklu_COX1_1_239	AGGGTTTGGT-AACTATTTAT
Skud_COX1_1_239	AGGTTTTGGT-AACTATTTAT
Scer_COX1_3_237	AGGTTTTGGT-AACTATTTAT
## 4	
Lmey_COX1_2_384	GATGAACTGT-ATATCCTCCT
Lthe_COX1_2_384	GATGAACTGT-ATATCCTCCT
Lklu_COX1_2_384	GATGAACTGT-ATATCCTCCT
Kser_COX1_2_384	GATGAACTGT-GTATCCTCCT
Ndel_COX1_2_384	GTTGAACTGT-ATATCCACCA
Cgla_COX1_2_384	GTTGAACTGT-ATATCCACCA
Sbay_COX1_2_384	GATGAACTGT-ATATCCACCA
Skud_COX1_2_384	GATGAACTGT-ATATCCACCA
Smik_COX1_3_383	GATGAACTGT-ATATCCACCA
Spar_COX1_2_384	GATGAACTGT-ATATCCACCA
## 5	
Ndel_COX1_3_706	CATTTGTTTT-GATTCTTCGG
Smik_COX1_4_705	CACTTATTTT-GATTCTTTGG
Lklu_COX1_3_706	CATTTATTCT-GATTCTTTGG
Sbay_COX1_3_706	CATTTATTTT-GATTCTTTGG
Skud_COX1_3_706	CATTTATTTT-GATTCTTTGG
## 6	
Scer_COX1_4_716	ATTCTTTGGT-CACCCTGAAG
Ccas_COX1_2_721	ATTTTTTGGT-CATCCCGAAG
Lmey_COX1_3_717	ATTCTTCGGT-CATCCAGAAG
Lthe_COX1_3_717	ATTCTTCGGT-CATCCAGAAG
Ldas_COX1_2_718	ATTCTTTGGT-CATCCAGAAG
Ncas_COX1_2_718	ATTCTTTGGT-CATCCAGAAG
## 7	
Lmey_COX1_4_896	AGATGCAGAT-ACTAGAGCAT

The flanking sequences of 7 COB introns	
## 1	
Kser_COB_1_200	AGTAGAACAT-ATCATGAGAG
## 2	
Lklu_COB_1_392	ATTTTTAGGT-TATTGTTGTG
Lmey_COB_1_392	TTTCTTAGGT-TATTGTTGTG
Sbay_COB_1_392	TTTCTTAGGT-TATTGTTGTG
Skud_COB_1_392	TTTTTTAGGT-TATTGTTGTG
Smik_COB_1_392	TTTTTTAGGT-TATTGTTGTG
Spar_COB_1_392	TTTTTTAGGT-TATTGTTGTG
## 3	
Skud_COB_2_413	TATGGACAGA-TGTCACATTG
Smik_COB_2_413	TATGGACAGA-TGTCACATTG
Spar_COB_2_413	TATGGACAGA-TGTCACATTG
Scer_COB_1_414	TATGGACAGA-TGTCACATTG
## 4	
Skud_COB_3_426	ACATTGAGGT-GCACTAGTTA
Smik_COB_3_426	ACATTGAGGT-GCACTAGTTA
Spar_COB_3_426	ACATTGAGGT-GCACTAGTTA
Scer_COB_2_427	ACATTGAGGT-GCACTAGTTA
## 5	
Lmey_COB_2_504	GAGGTGGGTT-CTCTGTATCA
Lklu_COB_2_504	GAGGAGGGTT-CTCAGTATCA
Skud_COB_4_502	GAGGTGGGTT-CTCAGTATCT
Smik_COB_4_502	GAGGTGGGTT-CTCAGTATCT
Spar_COB_4_502	GAGGTGGGTT-CTCAGTATCT
Scer_COB_3_503	GAGGTGGGTT-CTCAGTATCT
## 6	
Smik_COB_5_751	TACTTTAGGT-CATCCTGATA
Scer_COB_4_752	TACTTTAGGT-CATCCTGATA
## 7	
Ccas_COB_1_806	AGCTTCTATT-GTACCTGAAT
Smik_COB_6_801	AGCATCTATT-GTACCTGAAT
Scer_COB_5_802	AGCATCTATT-GTACCTGAAT

The flanking sequences of 1 21S_RNA introns	
## 1	
Lmey_21S_rRNA_1_2624	GCTAGGGATA-ACAGGGTAAT
Ldas_21S_rRNA_1_2648	GCTAGGGATA-ACAGGGTAAT
Lklu_21S_rRNA_1_2444	GCTAGGGATA-ACAGGGTAAT
Ncas_21S_rRNA_1_2724	GCTAGGGATA-ACAGGGTAAT
Kser_21S_rRNA_1_2439	GCTAGGGATA-ACAGGGTAAT
Smik_21S_rRNA_1_2680	GCTAGGGATA-ACAGGGTAAT
Spar_21S_rRNA_1_2588	GCTAGGGATA-ACAGGGTAAT
Scer_21S_rRNA_1_2715	GCTAGGGATA-ACAGGGTAAT

Ldas_COX1_3_897	AGATGCAGAT-ACTAGAGCAT
Sbay_COX1_4_896	AGATGCAGAT-ACTAGAGCCT
Ccas_COX1_3_900	AGATGTAGAT-ACTAGAGCTT
## **8**	
Lmey_COX1_5_967	CAGTTGATTA-GCACTTATTT
Ldas_COX1_4_968	CAGTTGATTA-GCACTTATTT
Skud_COX1_4_968	CTCATGATTA-GCTCTAATCT
Scer_COX1_5_967	CTCATGATTA-GCTCTAATCC
Ndel_COX1_4_968	CTCATGATTA-GCAACAATTT
Cgla_COX1_3_969	CTCATGATTA-GCAACAATTT
Smik_COX1_5_967	CTCATGATTA-GCGACAATCT
## **9**	
Kser_COX1_3_1104	TTTCCATGAT-ACATACTACG
Lmey_COX1_6_1101	ATTCCATGAT-ACTTACTATG
Sbay_COX1_5_1102	ATTCCACGAT-ACTTATTACG
Smik_COX1_6_1101	ATTCCACGAT-ACTTACTACG
Spar_COX1_3_1104	ATTCCACGAT-ACTTACTACG
Scer_COX1_6_1101	ATTCCACGAT-ACTTACTACG
## **10**	
Sbay_COX1_6_1126	GGACATTTCC-ATTATGTATT
Skud_COX1_5_1127	GGACATTTTC-ACTATGTATT
Smik_COX1_7_1125	GGACATTTTC-ACTATGTATT
Spar_COX1_4_1128	GGACATTTTC-ACTATGTATT
Scer_COX1_7_1125	GGACATTTTC-ACTATGTATT

Note: The number after a series of '#' represented the relative location of each intron in different yeasts. The flanking sequence was named according to 'Species_Gene_ID_Loc', where the 'ID' indicated the absolute location of the intron in corresponding gene and species, the 'Loc' indicated the insert location of the intron in the coding sequence. For example, 'Smik_COX1_1_168' represented the first intron of *cox1* gene in MT genome of *S.mikatae*, the intron inserted in the 168th nucleotide of coding sequence. The flanking sequence was showed according to "5' 10 bp exon sequence-3' 10 bp exon sequence", where the '-' represented the insert location of the intron in the coding sequence.
