# Supplementary material for: Mitochondrial genome evolution in the Saccharomyces sensu stricto complex
Source: PLoS One. 2017 Aug 16;12(8):e0183035. doi: 10.1371/journal.pone.0183035 (PMC5558958; doi:10.1371/journal.pone.0183035)
Supplement: S7 Table — We first calculated the amino-acid identity of intron ORFs in different linages. We then calculated the nucleotide identity for the introns without ORF. The rows with yellow back color were corresponding to Group II introns. The ‘-’ indicated the intron did not present in the linage, or only present in one species of the linage. (PDF) [file pone.0183035.s011.pdf]

S7 Table. The intron identity in four yeast linages

Introns	Intron amino-acid identity				Intron nucleotide identity (without ORF)			
	Lachancea	***C/N***	***K/N***	***SSS***	***Lachancea***	***C/N***	***K/N***	***SSS***
*cox1*_1	-	-	-	0.99	-	-	-	-
*cox1*_2	-	-	-	0.94	-	-	-	-
*cox1*_3	0.88	0.57	-	0.86	-	-	0.48	0.57
*cox1*_4	0.83	-	-	-	-	-	-	0.51
*cox1*_5	-	-	-	0.71	-	-	-	-
*cox1*_6	0.88	-	-	-	-	-	-	-
*cox1*_7	0.79	-	-	-	-	-	-	-
*cox1*_8	0.97	0.68	-	0.95	-	-	-	-
*cox1*_9	-	-	-	0.84	-	-	-	-
*cox1*_10	-	-	-	-	-	-	-	0.83
*cob*_1	-	-	-	-	-	-	-	-
*cob*_2	0.67	-	-	0.51	-	-	-	0.73
*cob*_3	-	-	-	-	-	-	-	0.83
*cob*_4	-	-	-	0.76	-	-	-	-
*cob*_5	0.95	-	-	0.99	-	-	-	-
*cob*_6	-	-	-	0.64	-	-	-	-
*cob*_7	-	-	-	-	-	-	-	0.62
*rnl*_1	-	-	-	-	0.55	-	0.63	0.77

Note: We firstly calculated the amino-acid identity of intron ORFs in different linages. We then calculated the nucleotide identity for the introns without ORF. The rows with yellow backcolor were corresponding to Group II introns. The '-' indicated the intron did not present in the linage, or only present in one species of the linage.
