# Supplementary material for: Mitochondrial genome evolution in the Saccharomyces sensu stricto complex
Source: PLoS One. 2017 Aug 16;12(8):e0183035. doi: 10.1371/journal.pone.0183035 (PMC5558958; doi:10.1371/journal.pone.0183035)
Supplement: S8 Table — All 35 genes were divided into seven syntenic orthologous blocks constituted by fourteen transcriptional units known in S. cerevisiae. For other SSS yeasts, we predicted transcription initiation site based on the motif WTATAAGTA. The predicted transcriptional units were similar to that in S. cerevisiae (data not shown). (PDF) [file pone.0183035.s012.pdf]

S8 Table. Seven blocks in mitochondria genome of *SSS*

Blocks	Transcriptional units	Genes
1	1.1	*rnl*<br>*tRNA-Thr2*
	1.2	*tRNA-Cys*<br>*tRNA-His*
	1.3	*tRNA-Leu*<br>*tRNA-Gln*<br>*tRNA-Lys*<br>*tRNA-Arg1*<br>*tRNA-Gly*<br>*tRNA-Asp*<br>*tRNA-Ser1*<br>*tRNA-Arg2*
	1.4	*tRNA-Ala*<br>*tRNA-Ile*<br>*tRNA-Tyr*<br>*tRNA-Asn*<br>*tRNA-Met1*
	1.5	*cox2*
2	2.1	*tRNA-Phe*<br>*tRNA-Val*<br>*cox3*
	2.2	*tRNA-Thr1*
3	3.1	*tRNA-Met2*
	3.2	*RPM1*<br>*tRNA-Pro*
4	4.1	*cox1*<br>*atp8*<br>*atp6*
5	5.1	*tRNA-Glu*<br>*cob*
6	6.1	*rns*<br>*tRNA-Trp*
7	7.1	*atp9*
	7.2	*tRNA-Ser2*<br>*var1*

Note: All 35 genes were divided into seven syntenic orthologs blocks constituted by fourteen transcriptional units known in *S. cerevisiae*. For other *SSS* yeasts, we predicted transcription initiation site based on the motif WTATAAGTA. The predicted transcriptional units were similar to that in *S. cerevisiae* (data not shown).
