# Supplementary material for: Mitochondrial genome evolution in the Saccharomyces sensu stricto complex
Source: PLoS One. 2017 Aug 16;12(8):e0183035. doi: 10.1371/journal.pone.0183035 (PMC5558958; doi:10.1371/journal.pone.0183035)
Supplement: S9 Table — The upper triangle of the table indicated the number of rearrangement events. The rearrangement scenarios were shown in supplement file (S1B File) in more detail. (PDF) [file pone.0183035.s013.pdf]

S9 Table. The number of rearrangement events between *SSS* yeasts

	S.cerevisiae	*S.paradoxus*	*S.mikatae*	*S.kudriavzevii*	*S.uvarum*
S.cerevisiae	0	2	1	2	3
S.paradoxus	-	0	3	3	4
S.mikatae	-	-	0	1	3
S.kudriavzevii	-	-	-	0	3
S.uvarum	-	-	-	-	0

Note: The upper triangle of the table indicated the number of rearrangement events. The rearrangement scenarios were showed in supplement file (S1B File) in more detail.
