# Supplementary material for: Mitochondrial genome evolution in the Saccharomyces sensu stricto complex
Source: PLoS One. 2017 Aug 16;12(8):e0183035. doi: 10.1371/journal.pone.0183035 (PMC5558958; doi:10.1371/journal.pone.0183035)
Supplement: S10 Table — The ‘Branch’ col indicated all evolutional branches in the phylogenetic tree of fifteen yeasts (S4 Fig). The ‘Leaf_Node’ col indicated the branches which included leaf nodes (i.e., fifteen yeasts). The ‘Branch Length’ col indicated the length of each branch in the phylogenetic tree. The ‘MT bsGOL’ indicated the branch-specific GOL of mitochondrial genome based on the pairwise comparisons of GOL. The ‘MT GOL Rate’ indicated the rearrangement rate of gene order in mitochondrial genome which was calculated by the ratio of bsGOL to branch length. The ‘Nuclear GOL Rate’ indicated the GOL rates in the nuclear genome of S. cerevisiae and S. paradoxus which were from Fischer et al. 2006 [30]. (PDF) [file pone.0183035.s014.pdf]

S10 Table. Branch-Specific Rates of GOL in fifteen yeasts

Leaf_Node	Branch	Branch Length	bsGOL	MT GOL Rate	Nuclear GOL Rate	MT/Nuclear rate
Scer	1	0.008116	0.04369	5.38	0.28	19.2
Spar	2	0.008054	0.07031	8.73	0.30	29.1
	3	0.003727	0.01802	4.84	……	……
Smik	4	0.007134	0.03948	5.53	……	……
	5	0.008487	0.00000	0.00	……	……
Skud	6	0.014752	0.04153	2.82	……	……
	7	0.016253	0.08952	5.51	……	……
Suva	8	0.016273	0.04550	2.80	……	……
	9	0.064636	0.19681	3.04	……	……
Kser	10	0.153053	0.23504	1.54	……	……
Ncas	11	0.131335	0.08896	0.68	……	……
	12	0.037610	0.03119	0.83	……	……
	13	0.018514	0.00000	0.00	……	……
Cgla	14	0.029167	0.00000	0.00	……	……
Ndel	15	0.033600	0.00000	0.00	……	……
	16	0.063384	0.19098	3.01	……	……
Ccas	17	0.111547	0.18458	1.65	……	……
Nbac	18	0.104545	0.37442	3.58	……	……
	19	0.054711	0.05902	1.08	……	……
	20	0.018655	0.00000	0.00	……	……
Lklu	21	0.036597	0.04955	1.35	……	……
Lthe	22	0.012066	0.00000	0.00	……	……
Ldas	23	0.014757	0.00000	0.00	……	……
Lmey	24	0.008364	0.00000	0.00	……	……
	25	0.008626	0.00000	0.00	……	……
	26	0.040301	0.03645	0.90	……	……
	27	0.081310	0.08169	1.00	……	……

Note: The ‘Branch’ col indicated all evolutional branches in the phylogenetic tree of fifteen yeasts (S4 Fig). The ‘Leaf_Node’ col indicated the branches which included leaf nodes (i.e., fifteen yeasts). The ‘Branch Length’ col indicated the length of each branch in the phylogenetic tree. The ‘MT bsGOL’ indicated the branch-specific GOL of mitochondrial genome based on the pairwise comparisons of GOL. The ‘MT GOL Rate’ indicated the rearrangement rate of gene order in mitochondrial genome which was calculated by the ratio of bsGOL to branch length. The ‘Nuclear GOL Rate’ indicated the GOL rates in the nuclear genome of *S. cerevisiae* and *S. paradoxus* which were from *Fischer et al.* 2006.
