# Supplementary material for: Mitochondrial genome evolution in the Saccharomyces sensu stricto complex
Source: PLoS One. 2017 Aug 16;12(8):e0183035. doi: 10.1371/journal.pone.0183035 (PMC5558958; doi:10.1371/journal.pone.0183035)
Supplement: S11 Table — We first predicted all nucleus tRNAs, rRNAs and RPR1 in other SSS yeasts based on the genes annotation in S. cerevisiae. Then we obtained the orthologous groups for all RNA genes by OrthoMCL [32]. After that, we calculated the nucleotide identity for each orthologous group, if the group included genes in all five SSS yeasts. (PDF) [file pone.0183035.s015.pdf]

S11 Table. The identity between nucleus and mitochondrial RNA genes

RNA Genes	Nucleus	Identity	Mitochondrial	Identity
RNase P	RPR1	0.77	RPM1	0.43
rRNA	RDN5	1.00		
	RDN18	0.99	15S_rRNA	0.84
	RDN25	0.98	21S_rRNA	0.75
	RDN58	1.00		
trnA	Ala1	1.00	Ala	0.96
	Ala2	1.00		
trnC	Cys	1.00	Cys	0.97
trnD	Asp	1.00	Asp	1.00
trnE	Glu	0.99	Glu	0.97
trnF	Phe1	1.00	Phe	1.00
	Phe2	1.00		
trnG	Gly1	1.00	Gly	0.99
	Gly2	1.00		
	Gly3	0.97		
	Gly4	1.00		
trnH	His	0.93	His	0.97
trnI	Ile1	1.00	Ile	0.97
	Ile2	1.00		
trnK	Lys1	1.00	Lys	0.96
	Lys2	1.00		
trnL	Leu1	1.00	Leu	0.99
	Leu2	1.00		
	Leu3	0.99		
trnM	Met1	1.00	Met1	1.00
	Met2	0.97	Met2	1.00
trnN	Asn	1.00	Asn	0.95
trnP	Pro1	1.00	Pro	0.99
	Pro2	1.00		
trnQ	Gln1	1.00	Gln	1.00
	Gln2	1.00		
trnR	Arg1	1.00	Arg1	0.99
	Arg2	1.00	Arg2	0.97
	Arg3	1.00		
trnS	Ser1	1.00		
	Ser2	1.00	Ser1	0.98
	Ser3	1.00	Ser2	0.99
	Ser4	0.96		
trnT	Thr1	0.97	Thr1	1.00
	Thr2	0.97	Thr2	0.97
	Thr3	1.00		
trnV	Val1	0.97	Val	0.97
	Val2	1.00		
	Val3	1.00		
trnW	Trp	1.00	Trp	0.95
trnY	Tyr	0.99	Tyr	0.98

Note: We first predicted all nucleus tRNAs, rRNAs and RPR1 in other *SSS* yeasts based on the genes annotation in *S. cerevisiae*. Then we obtained the orthologous groups for all RNA genes by OrthoMCL. After that, we calculated the nucleotide identity for each orthologous group, if the group included genes in all five SSS yeasts.
