# Supplementary material for: Mitochondrial genome evolution in the Saccharomyces sensu stricto complex
Source: PLoS One. 2017 Aug 16;12(8):e0183035. doi: 10.1371/journal.pone.0183035 (PMC5558958; doi:10.1371/journal.pone.0183035)
Supplement: S12 Table — The ‘Average’ rows in the table indicated the average of the dN/dS ratio of each mitochondria protein between two close relative species in the same lineage. The number in ‘Rank’ rows represent the rank number based on the ‘Average’. (PDF) [file pone.0183035.s016.pdf]

S12 Table. The dN/dS ratio for all linages

#Spe1-Spe2	COX1	COX2	COX3	ATP6	ATP8	ATP9	COB	VAR1
Lklu-Ldas	0.0802	0.1385	0.1557	0.2292	0.1384	0.0423	0.116	0.9799
Lmey-Ldas	0.0311	0.0657	0.1068	0.0893	0.1513	0.1085	0.0405	0.2859
Lmey-Lklu	0.0762	0.1199	0.1851	0.2824	0.0758	0.0664	0.1413	0.6666
Lthe-Ldas	0.077	0.0914	0.1372	0.0614	0.0726	0	0.1068	0.3431
Lthe-Lklu	0.092	0.0869	0.1872	0.2732	0.0431	0.0629	0.0782	0.7399
Lthe-Lmey	0.0489	0.0681	0.1304	0.0574	0	0.0816	0.1274	0.1941
**Average**	**0.0676**	**0.0951**	**0.1504**	**0.1655**	**0.0802**	**0.0603**	**0.1017**	**0.5349**
**Rank**	**7**	**5**	**3**	**2**	**6**	**8**	**4**	**1**
Ccas-Cgla	0.1799	0.4076	0.4017	0.6908	0.4332	0.2369	0.2734	1.1595
Nbac-Cgla	0.1779	0.4012	0.3379	0.6057	0.4255	0.2548	0.2768	1.136
Nbac-Ccas	0.1317	0.3139	0.4561	0.5677	0.4654	0.0758	0.2362	1.0978
Ndel-Cgla	0.0329	0.1104	0.1039	0.2053	0.0261	0.1022	0.0299	0.3713
Ndel-Ccas	0.1631	0.2714	0.4242	0.7253	0.1931	0.3607	0.2438	1.0531
Ndel-Nbac	0.1458	0.2737	0.3384	0.5631	0.3281	0.4796	0.2475	0.8887
**Average**	**0.1386**	**0.2964**	**0.3437**	**0.5597**	**0.3119**	**0.2517**	**0.2179**	**0.9511**
**Rank**	**8**	**5**	**3**	**2**	**4**	**6**	**7**	**1**
Ncas-Kser	0.1806	0.1414	0.3307	0.6486	0.5509	0.2389	0.2157	0.896
**Average**	**0.1806**	**0.1414**	**0.3307**	**0.6486**	**0.5509**	**0.2389**	**0.2157**	**0.8960**
**Rank**	**7**	**8**	**4**	**2**	**3**	**5**	**6**	**1**
Scer-Suva	0.0698	0.0652	0.0526	0.163	0.1344	0	0.0963	0.2887
Skud-Suva	0.0547	0.0735	0.0779	0.1636	0.1789	0	0.0842	0.882
Skud-Scer	0.0985	0.0474	0.0853	0.1853	0.2749	0	0.0658	0.5414
Smik-Suva	0.04	0.116	0.063	0.19	0.1344	0	0.0853	0.4383
Smik-Scer	0.0779	0.1504	0.0509	0.1738	0	0	0.1229	0.6015
Smik-Skud	0.0571	0.0585	0.0448	0.2061	0.2749	0	0.0277	0.4935
Spar-Suva	0.0557	0.116	0.0549	0.1652	0.1789	0	0.1258	0.3581
Spar-Scer	0.074	0.1113	0.0562	0.2735	0.2749	0	0.0748	0.661
Spar-Skud	0.0476	0.0421	0.06	0.1487	0	0	0.0849	0.5981
Spar-Smik	0.0316	0.0838	0.0757	0.2272	0.2749	0	0.1205	0.5408
**Average**	**0.0607**	**0.0864**	**0.0621**	**0.1896**	**0.1726**	**0.0000**	**0.0888**	**0.5403**
**Rank**	**7**	**5**	**6**	**2**	**3**	**8**	**4**	**1**

Note: The 'Average' rows in the table indicated the average of the dN/dS ratio of each mitochondria protein between two close relative species in the same lineage. The number in 'Rank' rows represent the rank number based on the 'Average'.
