# Supplementary material for: Mitochondrial genome evolution in the Saccharomyces sensu stricto complex
Source: PLoS One. 2017 Aug 16;12(8):e0183035. doi: 10.1371/journal.pone.0183035 (PMC5558958; doi:10.1371/journal.pone.0183035)
Supplement: S1 File — (A) Intergenic regions expansion. (B) The rearrangement scenarios in the SSS. (PDF) [file pone.0183035.s017.pdf]

S1 File.

(A) Intergenic regions expansion

Firstly, nine putative ORFs were found in the *SSS*. ORF1 is highly conserved among *S. kudriavzevii, S. mikatae, S. paradoxus* and *S. cerevisiae*. ORF5 is conserved within *S. paradoxus* and *S. cerevisiae*. Secondly, we found totally 24 *ori* sequences with the length about 0.26-2.3 kb in the *SSS*. The previous study conjectured that the increasing number of replication origins may lead to the change of MT genome size [1]. Thirdly, we predicted about 25-40 kb AT spacers in the *SSS* corresponded to about 60%-84% intergenic sequences, and the length increased about 10-15 times than those in *C. glabrata*. We calculated the (AT+TA)/(AA+TT) (R) ratios of the AT spacers for all fifteen yeasts [2] (S1C Fig). The *SSS* show similar R ratios (R = 1.53±0.08), but the R ratios in C/N linage (average: R = 2.4) were significantly larger than that in other linages (rank sum test, p = 0.0015). Fourthly, we investigated the GC clusters which comprise at least one G/C tetranucleotide (S2 Fig and S4-S5 Tables). Our results showed that the relative number of GC clusters are positively associated with the MT genome size (S1D Fig). Most GC clusters in *SSS* were related to the A, B, C clusters of *ori* sequences [3-6] (S3 Fig), which were apparently different from that in other linages (S5 Table). Although, the organization of GC clusters had a large diversity in different yeasts, all yeasts included about 10%~50% palindromic-like GC clusters, which can be folded into hairpin structures.

(B) The rearrangement scenarios in the *SSS*

S. cerevisiae-S. paradoxus

Compare to the *S. cerevisiae* mitochondrial genome, *S. paradoxus* showed a transposition of block 2 (*cox3*), block 3 (*rpm1*) and block 6 (*rns*) and an inversion of block 6 (*rns*).

S. cerevisiae-S. mikatae

Compare to the *S. cerevisiae* mitochondrial genome, *S. mikatae* showed a transposition of block 6 (*rns*).

S. cerevisiae-S. kudriavzevii

Compare to the *S. cerevisiae* mitochondrial genome, *S. kudriavzevii* showed a transpositions of block 6 (*rns*) and an inversion of block 7 (*atp9-trnS2-var1*).

S. cerevisiae-S. uvarum

Compare to the *S. cerevisiae* mitochondrial genome, *S. uvarum* showed a transpositions of block 6 (*rns*), a transposition of block 5 (*cob*) and an inversion of *var1-trnS2* in block 7 (*atp9-trnS2-var1*).

S. paradoxus-*S. mikatae*

Compare to the *S. paradoxus* mitochondrial genome, *S. mikatae* showed a transposition of block 4 (*cox1*-*atp8*-*atp6*) and block 5 (*cob*), a transposition of block 7 (*atp9*-*trnS2*-*var1*) and an inversion of block 6 (*rns*).

S. paradoxus-*S. kudriavzevii*

Compare to the *S. paradoxus* mitochondrial genome, *S. kudriavzevii* showed an inversion of block 2 (*cox3*), block 3 (*rpm1*), block 6 (*rns*) and block 7 (*atp9*-*trnS2*-*var1*) and an inversion of block 2 (*cox3*) and block 3 (*rpm1*) and a transposition of block 4 (*cox1*-*atp8*-*atp6*), block 5 (*cob*) and block 6 (*rns*).

S. paradoxus-*S. uvarum*

Compare to the *S. paradoxus* mitochondrial genome, *S. uvarum* showed a transposition of block 4 (*cox1*-*atp8*-*atp6*) and block 7 (*atp9*-*trnS2*-*var1*), a transposition block 5 (*cob*), an inversion of *var1*-*trnS2* in block 7 (*atp9*-*trnS2*-*var1*) and an inversion of block 6 (*rns*).

S. mikatae-*S. kudriavzevii*

Compare to the *S. mikatae* mitochondrial genome, *S. kudriavzevii* showed an inversion of block 7 (*atp9*-*trnS2*-*var1*).

S. mikatae-*S. uvarum*

Compare to the *S. mikatae* mitochondrial genome, *S. uvarum* showed a transposition of block 5 (*cob*), an inversion of *var1*-*trnS2* in block 7 (*atp9*-*trnS2*-*var1*) and a transpositions of of block 6 (*rns*).

S. kudriavzevii-*S. uvarum*

Compare to the *S. kudriavzevii* mitochondrial genome, *S. uvarum* showed a transposition of block 3 (*rpm1*) and block 4 (*cox1*-*atp8*-*atp6*), an inverse transposition of *atp9* in block 7 (*atp9*-*trnS2*-*var1*) and a transposition of block 6 (*rns*).

References

1. Goursot R, Mangin M, Bernardi G: **Surrogate origins of replication in the mitochondrial genomes of ori-zero petite mutants of yeast**. *Embo Journal* 1982, **1**(6):705-711.
2. de Zamaroczy M, Bernardi G: **The AT spacers and the var1 genes from the mitochondrial genomes of Saccharomyces cerevisiae and Torulopsis glabrata: evolutionary origin and mechanism of formation**. *Gene* 1987, **54**(1):1-22.
3. Zamaroczy MD, Bernardi G: **The GC clusters of the mitochondrial genome of yeast and their evolutionary origin**. *Gene* 1986, **41**(1):1-22.
4. Bernardi G: **Lessons from a small, dispensable genome: the mitochondrial genome of yeast**. *Gene* 2005, **354**:189-200.
5. Solieri L: **Mitochondrial inheritance in budding yeasts: towards an integrated understanding**. *Trends in microbiology* 2010, **18**(11):521-530.
6. Sanchez-Sandoval E, Diaz-Quezada C, Velazquez G, Arroyo-Navarro LF, Almanza-Martinez N, Trasviña-Arenas CH, Brieba LG: **Yeast mitochondrial RNA polymerase primes mitochondrial DNA polymerase at origins of replication and promoter sequences**. *Mitochondrion* 2015, **24**:22-31.
